# Differential Requirement of *Cd8* Enhancers E8_I_ and E8_VI_ in Cytotoxic Lineage T Cells and in Intestinal Intraepithelial Lymphocytes

**DOI:** 10.3389/fimmu.2019.00409

**Published:** 2019-03-11

**Authors:** Alexandra Franziska Gülich, Teresa Preglej, Patricia Hamminger, Marlis Alteneder, Caroline Tizian, Maria Jonah Orola, Sawako Muroi, Ichiro Taniuchi, Wilfried Ellmeier, Shinya Sakaguchi

**Affiliations:** ^1^Division of Immunobiology, Institute of Immunology, Center for Pathophysiology, Infectiology and Immunology, Medical University of Vienna, Vienna, Austria; ^2^Laboratory for Transcriptional Regulation, RIKEN Center for Integrative Medical Sciences (IMS), Yokohama, Japan

**Keywords:** T cell development, gene regulation, CD8, enhancer, transgenic/knockout mice, cytotoxic T cells, IELs, CD4 CTLs

## Abstract

CD8 expression in T lymphocytes is tightly regulated by the activity of at least six *Cd8* enhancers (E8_I_-E8_VI_), however their complex developmental stage-, subset-, and lineage-specific interplays are incompletely understood. Here we analyzed ATAC-seq data on the Immunological Genome Project database and identified a similar developmental regulation of chromatin accessibility of a subregion of E8_I_, designated E8_I_-core, and of E8_VI_. Loss of E8_I_-core led to a similar reduction in CD8 expression in naïve CD8^+^ T cells and in IELs as observed in *E8*_*I*_^−/−^ mice, demonstrating that we identified the core enhancer region of E8_I_. While *E8*_*VI*_^−/−^ mice displayed a mild reduction in CD8 expression levels on CD8SP thymocytes and peripheral CD8^+^ T cells, CD8 levels were further reduced upon combined deletion of E8_I_-core and E8_VI_. Moreover, activated *E8*_*I*_*-*core^−/−^*E8*_*VI*_^−/−^ CD8^+^ T cells lost CD8 expression to a greater degree than *E8*_*I*_*-*core^−/−^ and *E8*_*VI*_^−/−^ CD8^+^ T cells, suggesting that the combined activity of both enhancers is required for establishment and maintenance of CD8 expression before and after TCR activation. Finally, we observed a severe reduction of CD4 CTLs among the TCRβ^+^CD4^+^ IEL population in *E8*_*I*_*-*core^−/−^ but not *E8*_*VI*_^−/−^ mice. Such a reduction was not observed in *Cd8a*^−/−^ mice, indicating that E8_I_-core controls the generation of CD4 CTLs independently of its role in *Cd8a* gene regulation. Further, the combined deletion of E8_I_-core and E8_VI_ restored CD4 CTL subsets, suggesting an antagonistic function of E8_VI_ in the generation of CD4 CTLs. Together, our study demonstrates a complex utilization and interplay of E8_I_-core and E8_VI_ in regulating CD8 expression in cytotoxic lineage T cells and in IELs. Moreover, we revealed a novel E8_I_-mediated regulatory mechanism controlling the generation of intestinal CD4 CTLs.

## Introduction

CD8 plays an important role in the activation of cytotoxic T cells by serving as a coreceptor for MHC class I-restricted T cell receptors via its binding to the invariant α3 domain of MHC class I (1). The expression of the CD8 coreceptor is therefore closely linked with the development and function of the cytotoxic T cell lineage and has to be tightly controlled (2, 3). Whereas, double-positive (DP) thymocytes, CD8 single-positive (SP) and almost all peripheral conventional cytotoxic T cells express CD8 as a heterodimer consisting of the CD8α and CD8β chains (encoded by the closely linked *Cd8a* and *Cd8b1* genes), some subsets of intraepithelial lymphocytes (IELs) in the gut (4, 5) and CD8^+^ dendritic cells (DCs) (6) express CD8 as a CD8αα homodimer. Moreover, a fraction of activated cytotoxic T cells upregulates *Cd8a* gene expression, leading to the formation of CD8αα in addition to CD8αβ heterodimers (7). Therefore, both genes are coordinately as well as independently regulated in different cell lineages and T cell subsets. The dynamic and complex pattern of CD8 expression is regulated by at least six *Cd8* enhancers, designated E8_I_ to E8_VI_, located within the *Cd8ab* gene complex. A series of transgenic reporter gene expression assays as well as the analyses of mice harboring single and combinatorial deletion of *Cd8* enhancers revealed developmental stage-, lineage-, and subset-specific activities of these enhancers. Together, these studies revealed a highly complex and partially also synergistic network of *cis*-regulatory elements driving CD8 expression (8–10).

Among the *Cd8* enhancers identified, E8_I_ is the most intensively studied enhancer. E8_I_ directs expression in cytotoxic lineage cells (i.e., mature CD8 SP thymocytes and cytotoxic T cells) as well as in CD8αα^+^ and CD8αβ^+^ IELs in the gut (11, 12). In line with its enhancer activity in IELs, the analysis of *E8*_*I*_^−/−^ mice revealed a severe reduction in CD8αα expression on *E8*_*I*_^−/−^ IELs, particularly in CD8αα^+^TCRγδ^+^ IELs (13, 14). In contrast, there is normal CD8 expression in *E8*_*I*_^−/−^ cytotoxic lineage cells, except of a mild reduction of CD8 expression in mature CD8SP thymocytes, suggesting compensatory mechanisms by other *Cd8* enhancer(s) (13, 14). Subsequent studies revealed additional important roles for E8_I_ in the regulation of CD8 expression and hence also in the control of T cell effector function. It was shown that cytotoxic T cells start to express CD8αα homodimers on their surface (in addition to CD8αβ heterodimer) upon viral and bacterial infection (7, 15–17). The upregulation of *Cd8a* gene expression leading to CD8αα homodimer formation, which was postulated to be required for the generation of memory cytotoxic T cells, is largely mediated by E8_I_ (7, 15). Moreover, we demonstrated that E8_I_ is required for the maintenance of *Cd8a* expression during T cell activation, in part by epigenetic programing of the *Cd8ab* gene complex and via Runx3 recruitment, since activated *E8*_*I*_^−/−^ cytotoxic T cells downmodulate CD8 expression, leading to impaired effector function (18). In addition to its important role in CD8 lineage T cells, E8_I_ functions unexpectedly also in CD4 lineage T cells. While conventional CD4^+^ T cells express high levels of ThPOK and low levels of Runx3 (3, 10), a fraction of intestinal intraepithelial CD4^+^ helper T cells displays a ThPOK^lo^Runx3^hi^ transcription factor expression pattern. This is accompanied with the upregulation of cytotoxic features, such as the expression of CD8αα homodimers, Granzyme B, CD103 and 2B4 proteins (19, 20). It was shown that the induction of CD8αα expression in these CD4 CTLs is largely dependent on the activity of E8_I_ (20). Further, CD4^+^ T cells lacking HDAC1 and HDAC2 upregulate several cytotoxic features including CD8, and the upregulation of CD8 is also dependent on E8_I_ (21). Thus, while CD8 expression is largely dependent on E8_I_ in activated/effector T cells as well as in IELs, the *Cd8* enhancers essential for CD8 expression in naïve CD8^+^ T cells and/or that compensate for loss of E8_I_ have not been identified. Moreover, E8_I_-deficient mice harbor a deletion of a 7.6 kb genomic region (13, 14) and it is not known whether the various activities of E8_I_ in CD8^+^ T cells as well as in CD4 CTLs reside within the same regions of the larger genomic fragment.

In this study we revisited the *Cd8ab1* gene complex and analyzed publically available ATAC-seq data on the Immunological Genome Project (ImmGen) database (22). This revealed a similar developmental regulation and opening of chromatin accessibility in mature CD8^+^ T cells of a subregion within E8_I_ (designated E8_I_-core) and of *Cd8* enhancer E8_VI_, which displays also enhancer activity in mature cytotoxic T cells (23). Transgenic reporter gene expression assays with a 554bp fragment containing E8_I_-core demonstrated a similar enhancer activity as shown for the large genomic E8_I_ fragment. To test the potential interplay between E8_I_-core and E8_VI_, we generated E8_I_-core, E8_VI_, and E8_I_-core/E8_VI_-doubly-deficient mice. Our data revealed that *E8*_*I*_*-*core^−/−^ mice “phenocopied” the alterations in CD8 expression in the cytotoxic lineage and in intestinal IELs as observed in *E8*_*I*_^−/−^ mice, while activated E8_I_-core-deficient CD8^+^ T cells maintained CD8 expression to a greater extent than E8_I_-deficient CD8^+^ T cells. This suggests the existence of an additional regulatory element in addition to E8_I_-core that functions in activated CD8^+^ T cells within E8_I_. *E8*_*VI*_^−/−^ mice displayed a mild reduction in CD8 expression levels on CD8SP thymocytes and peripheral CD8^+^ T cells, while CD8α expression levels in IELs remained unchanged in the absence of E8_VI_. Compared to single E8_I_-core and E8_VI_ mutant mice, the combined deletion of both E8_I_-core and E8_VI_ led to a further reduction of CD8 expression in cytotoxic lineage cells. Moreover, anti-CD3/CD28-stimulated *E8*_*I*_*-*core^−/−^*E8*_*VI*_^−/−^ CD8^+^ T cells down-modulated CD8 expression to a greater degree than *E8*_*I*_*-*core^−/−^ and *E8*_*VI*_^−/−^ CD8^+^ T cells, suggesting that the combined activity of both enhancers is required for establishment and maintenance of CD8 expression before and after TCR activation. Finally, *E8*_*I*_*-*core^−/−^ but not *E8*_*VI*_^−/−^ TCRβ^+^CD8β^−^CD4^+^ IELs displayed a severe reduction in the percentages of the ThPOK^lo^Runx3^hi^ subset, characteristic for cytotoxic CD4^+^ T cells (CD4 CTLs). Such a reduction was not seen in *Cd8a*^−/−^ mice, indicating that E8_I_-core controls the generation of CD4 CTLs, independently of its role in *Cd8a* gene regulation. Of note, the combined deletion of both E8_I_-core and E8_VI_ led to the appearance of CD4 CTLs with a similar frequency as observed in WT mice, suggesting an antagonistic interplay between E8_I_-core and E8_VI_ in the generation of CD4 CTLs. Together, our study genetically demonstrates that CD8 expression in cytotoxic lineage T cells and IELs is directed by a complex utilization and interplay of E8_I_-core and E8_VI_. Moreover, our data indicate a novel role for E8_I_ in regulating the differentiation of CD4 CTLs in the gut.

## Materials and Methods

### Mice

ECR-8 transgenic mice were generated at the Japan SLC, Inc. (Hamamatsu-shi, Shizuoka, Japan), and *E8*_*I*_-*core*^−/−^, *E8*_*VI*_^−/−^, and *E8*_*I*_-*core*^−/−^*E8*_*VI*_^−/−^ were generated at the Animal Facility Group at the RIKEN IMS (Yokohama, Japan). *E8*_*I*_^−/−^ (13), *Cd8a*^−/−^ (24), *E8*_*I*_-Cre (25), Rosa26-stop-YFP reporter (26) mice have been described previously. Mice used for experiments were 6–10 weeks old and were maintained in the preclinical research facility of the Medical University of Vienna and in the animal facility of the RIKEN IMS. Animal husbandry and experimentation was performed under the national laws (Federal Ministry for Science and Research, Vienna, Austria) and ethics committees of the Medical University of Vienna and according to the guidelines of FELASA, which match that of ARRIVE. Animal husbandry and experimentation at the RIKEN IMS was approved by IACUC of RIKEN Yokohama Branch.

### Generation of Transgenic Mice

The basic *Cd8a* promoter-human CD2 (hCD2) reporter construct was previously described (11). The E8_I_-core fragment was amplified by PCR, and subcloned into EcoRI and HindIII sites upstream of the *Cd8a* promoter. The following primers were used for PCR (the EcoRI site was added for cloning purposes, whereas the HindIII site was encoded in endogenous *Cd8ab* gene complexes. These restriction sites are underlined): E8_I_core-F: 5′- TAGAATTCGGCTACCTCTGTCTCCC-3′ and E8_I_core-R: 5′- TATGGATCCAAGCTTGTGAATGGACCACTGAG-3′. Eggs from C57BL/6 mice were injected with the transgenic construct according to standard procedures. Transgenic founders were identified by PCR and either analyzed or backcrossed onto the C57BL/6 background. A total of 11 founders were generated, of which 5 expressed the hCD2 reporter gene. Transgenic lines #1 and #2 were generated from two founders (founders 1–3 and 1–1, respectively).

### Generation of *Cd8* Enhancer-Deficient Mice

pBluescript (pBS: Startagene) plasmids harboring various genomic fragments from the murine *Cd8a* and *Cd8b1* loci (11) were used as template for PCR amplifications during the construction of the targeting vector. E8_I_-core region (to which a loxP site was added at the 5′ end) and part of the long arm were PCR amplified, and were ligated using an additionally generated EcoRI site. A 5.6 kb BamHI/FspI fragment was cut out from pWE216 plasmid harboring E8_I_ and surrounding genomic regions (unpublished), and was inserted upstream of the aforementioned DNA sequence. The short arm (to which XhoI and KpnI/XbaI sites were added at the 5′ and 3′ end, respectively) was PCR amplified, and was ligated into the XhoI and XbaI sites of pL2Neo2 plasmid containing the neomycin resistance gene cassette (*Neo*^*r*^) flanked by two loxP sites (floxed) (27). Finally, a BamHI/SalI fragment (harboring the long arm, a loxP site and the E8_I_-core region) and a SalI/KpnI fragment (harboring the floxed *Neo*^*r*^ and the short arm) were inserted into pBS by tri-molecular ligation. The targeting vector was linearized by SacII digestion and was transfected into the M1 ES cell line as previously described (28). Homologous recombination in ES cells was screened by PCR with primers indicated in Figure S2. The aggregation of ES cell clone was performed as previously described (28). Subsequently, mice with the targeted allele were bred with CMV-Cre transgenic mice to delete the *Neo*^*r*^. The genotyping of *E8*_*I*_-core^−/−^ mice was carried out by PCR using the following three primers: E8_I_-Lox5: 5′-TTCCCATGAGGAACAGAGCTGG-3′, E8_I_-core F1: 5′-GACCTGACTTAACCTATGAGTGG-3′ and E8_I_-D3-3: 5′-CCATACTCAGCTTCTGACTCTCTGGC-3′ (the wild-type allele: 214 bp, the deleted allele: 301 bp). *E8*_*VI*_^−/−^ and *E8*_*I*_-core^−/−^*E8*_*VI*_^−/−^ mice were generated using the CRISPR/Cas9 system. *Cas9* mRNA and the following guide RNAs were injected into the cytoplasm of C57BL/6 as well as *E8*_*I*_-core^−/−^ fertilized eggs as previously described (29): E8_VI_-gRNA-5: 5'-CAGCCCUGAGCUGACAUUCAUGG-3′ and E8_VI_-gRNA-3: 5′-UCUGAGUUUAAGCAGCAGUGUGG-3′. Resultant offspring were screened by PCR using the following primers: E8_VI_-F: 5′-CCATCAGGTACTTGGGAATGCTCAG-3′ and E8_VI_-R: 5′-CACAAAGTAGATCACAGGATATGGG-3′, and the successful deletion of E8_VI_ was confirmed by sequencing. Mice carrying the desired mutation were bred with C57BL/6 mice to confirm germline transmission, and were subsequently intercrossed to obtain *E8*_*VI*_^−/−^ and *E8*_*I*_-core^−/−^*E8*_*VI*_^−/−^ mice. The genotyping PCR was performed using E8_VI_-F and E8_VI_-R primers (the wild-type allele: 749 bp, the deleted allele: 225 bp).

### Cell Preparation

Single cell suspensions of thymocytes and splenocytes were prepared as previously described (30). DCs were isolated according to a published protocol with minor modifications (31). In brief, spleens were injected with RPMI 1640 medium (Sigma) containing 600 U/ml Collagenase D (Roche), 20 U/ml DNase I (Roche) and 20 mM HEPES (Sigma), and cut into small pieces using sterile scissors. Subsequently, spleen samples were incubated in 5 ml of the same RPMI 1640 medium at 37°C for 30 min at 180 rpm in a shaker. Splenocytes were pushed through a 70 μm cell strainer (BD Biosciences), suspended in 2 ml of Lymphoprep (STEMCELL technologies) and centrifuged at 1,700 rpm for 15 min at room temperature. Cells at the low-density fraction were isolated and stained with appropriate antibodies. For the stimulation of DCs, low-density cells were incubated in 1 ml of complete RPMI1640 medium [Sigma, supplemented with 10% FCS (Sigma), 100 U/ml penicillin-streptomycin (GE Healthcare), 2 mM L-glutamin (Sigma), 0.1 mM non-essential amino acid (Lonza), 1 mM sodium pyruvate (GE Healthcare), 55 μM of β-mercaptoethanol (Sigma)] containing 500 ng/ml Lipopolysaccharide (LPS) (InvivoGen) at 37°C for 24 h. For most of the experiments IELs were isolated as previously described (11). In brief, small intestines were removed from the peritoneum of euthanized mice and the gut lumen was flushed with RPMI 1640 medium supplemented with 2% FCS. The intestine was turned “inside-out” over a polyethylene tube and incubated in 50 ml of RPMI supplemented with 10% FCS and 20 mM HEPES at 37°C for 1 h at 100 rpm in a shaker to release IELs into the medium. IELs were centrifuged at 1,700 rpm for 5 min at room temperature, suspended in RPMI 1640/2% FCS medium containing 37% Percoll (GE Healthcare) and were centrifuged at 1,700 rpm for 30 min at room temperature. Subsequently, cells were suspended in BD Pharm Lyse buffer (BD Biosciences) to remove red blood cells, washed with PBS/2% FCS and stained with the appropriate antibodies. To examine *Cd8a* gene expression in TCRβ^+^CD8β^−^CD4^+^ IELs, IELs were isolated by collagenase digestion (20). Briefly, small intestines were isolated, Peyer's batches were removed and tissue was cut into small pieces. The tissue pieces were incubated with HBSS buffer (Sigma) supplemented with 5 mM EDTA (Sigma) at 37°C for 15 min at 200 rpm in a shaker. Subsequently, cells were pelleted and further digested with HBSS buffer supplemented with 100 U/ml collagenase D at 37°C for 30 min at 200 rpm in a shaker. After digestion cells were resuspended in HBSS buffer containing 40% Percoll, layered over HBSS/80% Percoll and centrifuged at room temperature for 30 min at 2,000 rpm. Cells from the 40/80% interface were collected, washed and resuspended in PBS/2% FCS. CD19^−^TCRγδ^−^TCRβ^+^CD8β^−^CD4^+^ IEL subset was sorted with a SH800S Cell Sorter (Sony Biotechnology) and used for subsequent gene expression analysis.

### Isolation and Activation of CD4^+^ and CD8^+^ T Cells

CD4^+^ and CD8^+^ T cells were first enriched by negative depletion before cell sorting. In brief, after red blood cell lysis, splenocytes (5–10 × 10^7^ cells) were incubated with biotinylated (bio)-anti-Gr1 (RB6-8C5, final concentration 4 μg/ml), bio-anti-CD45R (RA3-6B2, 4 μg/ml), bio-anti-Ter119 (Ter119, 1 μg/ml), bio-anti-NK1.1 (PK136, 1 μg/ml), bio-anti-CD11b (M1/70, 1 μg/ml), bio-anti-CD11c (HL3, 1 μg/ml), bio-anti-CD8α (53–6.7, 2 μg/ml, for CD4^+^ T cell enrichment) and bio-anti-CD4 (RM4-5, 3μg/ml, for CD8^+^ T cell enrichment) in 0.5 ml PBS/2% FCS for 30 min at ice. The biotinylated antibodies were purchased from Biolegend and BD Biosciences. Subsequently, cells were washed and purified by negative depletion using streptavidin beads (BD Biosciences) according to the manufacturer's protocol. Enriched CD4^+^ and CD8^+^ T cells were sorted with a SH800S Cell Sorter for the CD4^+^CD8α^−^CD62L^+^CD44^−^CD25^−^ and CD4^−^CD8α^+^CD62L^+^CD44^−^ populations, respectively. Sorted naïve CD4^+^ and CD8^+^ T cells were stained with Cell Proliferation Dye eFluor 450 (Thermo Fisher Scientific) according to the manufacturer's protocol, and were stimulated (0.3–0.5 × 10^6^ cells/well) with plate-bound anti-CD3ε (145-2C11, 2 μg/ml; BD Biosciences) and anti-CD28 (37.51, 2 μg/ml; BD Biosciences) on 48 well plates in the presence of rhIL-2 (20 U/ml: Peprotech). CD8^+^ T cell cultures were split 1:2 48 h after activation, and cells were cultured for additional 24 h in the presence of 100 U/ml rhIL-2. For the treatment of CD4^+^ T cells with HDAC inhibitor, either MS-275 (Selleck Chemicals, used at a final concentration of 10 μM) or DMSO (as a carrier control) was added to CD4^+^ T cell culture 24 h after activation, and cells were cultured for additional 24 h.

### Antibodies and Flow Cytometry

Antibodies used in this study are listed in Table S1. Thymocytes, splenocytes, IELs and activated T cells were first incubated with Fixable Viability Dye eFluor 506 (Thermo Fisher Scientific) as well as purified anti-CD16/CD32 antibody (BD Biosciences) to avoid unspecific antibody binding. Subsequently, cells were incubated with appropriate antibodies against surface markers on ice for 30 min. For the intracellular staining of transcription factors Foxp3/Transcription Factor Staining Buffer Kit (Thermo Fisher Scientific) was used according to manufacturer's instructions. Intracellular ThPOK and Runx3 expression was detected either by Alexa Fluor 647 anti-mouse Zbtb7b (T43-94) and PE anti-Runx3 (R3-5G4: BD Biosciences) antibodies or by anti-ThPOK (D9V5T: Cell Signaling Technology) and anti-Runx3 (R3-5G4: BD Biosciences) antibodies, followed by Alexa Fluor 647 anti-mouse IgG1 (RMG1-1: Biolegend) and PE anti-rabbit IgG (H+L) (#8885, Cell Signaling Technology) antibody staining, respectively. Flow cytometric data were collected with LSRII or Fortessa (BD Biosciences), and were analyzed with Flowjo software (Treestar).

### cDNA Synthesis and Quantitative Real-Time PCR (qRT-PCR)

Total RNA was isolated using RNeasy Kits (Quiagen) according to manufacturer's instructions. RNA was reverse-transcribed using SuperScript III Reverse Transcriptase and Oligo(dT)18 Primer (Thermo Fisher Scientific). The majority of qRT-PCR was performed using iTaq Universal SYBR Green Supermix on the CFX 96 Real-Time PCR detection system (Bio-Rad). Primer pairs to detect *Cd8a, Cd8b1*, and *Hprt* gene expression were previously described (21). For the detection of *Cd8a* gene expression in TCRβ^+^CD8β^−^CD4^+^ IELs, TaqMan gene expression assays were performed using probes for *Cd8a* (Mm01182107_g1) and *Hprt* (Mm01182107_g1) genes (Thermo Fisher Scientific).

### Analysis of Publically Available ATAC-Seq Data

ATAC-seq data of the ImmGen database (22) were directly downloaded from Gene Expression Omnibus database (GEO accession: GSE100738). For the analysis of ATAC-seq data of TCRγδ^+^ IELs (GEO accession: GSE89646) (32), raw sequencing reads were downloaded from NCBI SRA database, and were retrieved using SraTailor software package (33).

### Statistical Analysis

The statistical analyses were performed using Prism 6 software (GraphPad). As indicated in each figure legend, *p*-values were calculated with either an unpaired Student's *t*-test, a one-sample *t*-test or a one-way ANOVA analysis followed by Tukey's multiple-comparison test. The *p*-values were defined as following: ^*^, *p* < 0.05; ^**^, *p* < 0.01; ^***^, *p* < 0.001. Differences that did not reach a statistically significant level (i.e., *p* ≥ 0.05) were either indicated as “n.s.” for two group comparisons or not indicated for multiple group comparisons.

## Results

### Evolutionary Conserved Regions at the *Cd8ab1* Gene Complex Overlap With Open Chromatin Regions in Cytotoxic Lineage Cells

**Figure 1:**
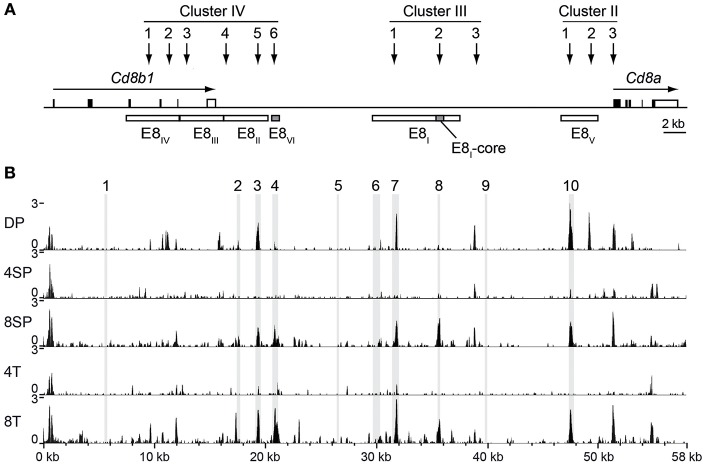
Chromatin accessibility at the *Cd8ab1* gene complex in the T cell lineage. **(A)** Schematic map of the *Cd8a* and *Cd8b1* gene loci [after Gorman et al. (38)]. Horizontal arrows indicate the transcriptional orientation of the *Cd8a* and *Cd8b1* genes. Vertical arrows indicate the localization of DNase I hypersensitivity sites that constitute clusters II, III, and IV (11, 36). The boxes below the *Cd8ab1* gene complex indicate the location of *Cd8* enhancers E8_I_ to E8_VI_. The E8-core and E8_VI_ regions are indicated as shaded boxes. **(B)** UCSC genome browser snapshots showing the ATAC-seq signals at the *Cd8ab1* gene complex (GRCm38/mm10, chr6: 71322001-71380000) in double-positive (DP), CD4 single-positive (4SP), CD8 single-positive (8SP) thymocytes, CD4^+^ T (4T) and CD8^+^ T (8T) cells. The ATAC-seq data were obtained from the Immunological Genome Project (ImmGen) (22). Shaded bars indicate the location of ECR1 to ECR10 as previously described (23).

Our previous studies demonstrated compensatory mechanisms between developmental stage-specific *Cd8* enhancers E8_I_ and E8_II_ (34) and E8_II_ and E8_III_ (35) in the regulation of CD8 expression at various stages of T cell development. However, E8_I_,E8_II_-doubly-deficient CD8SP thymocytes and naïve CD8^+^ T cells still express ~70% of CD8 levels compared to WT CD8^+^ T cells (34), suggesting that other (unknown) *Cd8* enhancer(s) are active in these subsets. In order to obtain additional insight into the complex regulation of CD8 expression during T cell development, we searched in the ImmGen ATAC-seq database (22) for developmental stage-specific open chromatin regions at the *Cd8ab1* loci. As expected, the ATAC-seq peaks nicely overlapped with previously identified DNase I hypersensitive sites at the *Cd8ab1* gene loci (11, 36) (Figures 1A,B). Moreover, five ATAC-seq peaks mapped to the evolutionary conserved regions (ECR)−3, −4, −7, −8, and −10, respectively (Figure 1B), which we have identified in a previous study (23) using the MULAN algorithm (37). Interestingly, two ATAC-seq peaks that overlap with ECR-8 and ECR-4 show a similar developmental regulation and appeared only in CD8SP thymocytes and CD8^+^ T cells (Figure 1B). Indeed, ECR-8 and ECR-4 mapped within E8_I_ and E8_VI_, respectively, both of which display enhancer activity in mature CD8SP thymocytes and in naive CD8^+^ T cells (11, 12, 23). Thus, ATAC-seq analysis revealed a strong correlation between enhancer activity and the chromatin status of E8_I_ and E8_VI_, suggesting that part of E8_I_ (i.e., ECR-8) and E8_VI_ (i.e., ECR-4) might synergistically regulate CD8 expression once the cytotoxic lineage has been specified.

### ECR-8 Represents the Core Enhancer Region of E8_I_

The E8_I_ enhancer activity has been initially identified within a 7.6 kb genomic fragment (11, 12) and subsequently mapped to a 1.6 kb genomic sub-fragment (13) that displayed identical enhancer activity (Figure S1A). Since ECR-8 is located within the 1.6 kb genomic sub-fragment and becomes accessible in cytotoxic lineage cells (Figure S1A), we performed transgenic reporter expression assays to test whether ECR-8 displays enhancer activity. A 544 bp fragment containing ECR-8 and the downstream open chromatin region was inserted into the previously generated basic reporter expression construct harboring the minimal *Cd8a* promoter (*P8a*) and a human *CD2 (hCD2)* reporter gene (Figures S1A,B and Table S2). From 11 transgenic founders identified, 5 displayed expression in CD8^+^ peripheral blood T lymphocytes, but none of these 5 founders displayed expression in CD4^+^ PBLs (data not shown). A more detailed analysis of 2 transgenic founders revealed that ECR-8 directed transgene expression in mature CD8SP thymocytes, in CD8^+^ T cells and in CD8αα^+^ IEL (Figures S1C–E). Thus, ECR-8 displays a similar activity as the initially described 7.6 kb genomic E8_I_ enhancer (11, 12) and the 1.6 kb genomic sub-fragment of E8_I_ (13). This suggests that ECR-8 represents the core enhancer region of the E8_I_ (hereafter designated as E8_I_-core, see also Figure 1A).

**Figure 2:**
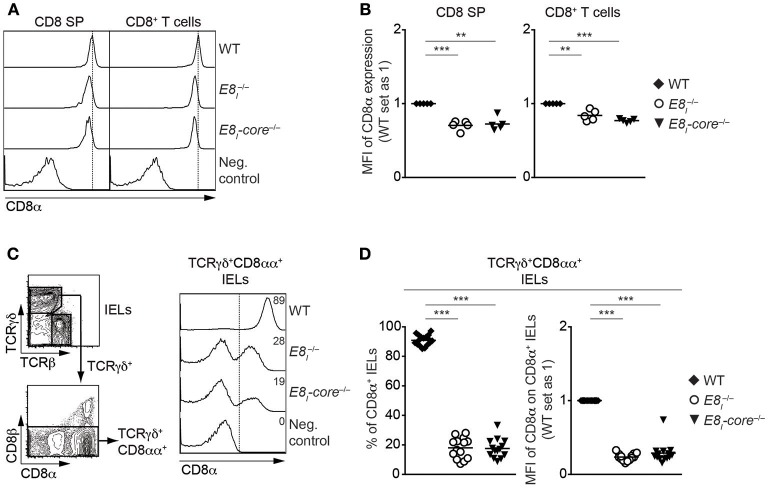
E8_I_-core represents the core enhancer region of E8_I_. **(A)** Histograms showing CD8α expression on CD8SP thymocytes and splenic CD8^+^ T cells isolated from wild-type (WT), *E8*_*I*_^−/−^ and *E8*_*I*_*-*core^−/−^ mice. The gating strategy is shown in Figures S3A,C. Dotted vertical lines indicate the peaks of CD8α expression on WT cells. CD8α expression on WT CD4SP thymocytes (left panel) or CD4^+^ T cells (right panel) is shown as negative staining control. **(B)** Diagrams showing the relative mean fluorescence intensity (MFI) of CD8α expression on CD8SP thymocytes (left) and splenic CD8^+^ (right) T cells isolated from wild-type (WT), *E8*_*I*_^−/−^ and *E8*_*I*_*-*core^−/−^ mice. **(C)** Representative gating strategy for the analysis of CD8α expression on TCRγδ^+^CD8αα^+^ IELs (left panel) and histograms showing CD8α expression on TCRγδ^+^CD8αα^+^ IELs isolated from wild-type (WT), *E8*_*I*_^−/−^ and *E8*_*I*_*-*core^−/−^ mice (right panel). CD8α expression on WT CD19^+^ B cells is shown as a negative staining control. Dotted lines and numbers indicate gating region for the CD8α^+^ population and the percentages of the CD8α^+^ population, respectively. **(D)** Diagrams showing the percentage of the CD8α^+^ population (left panel) and MFI CD8α expression levels (right panel) within TCRγδ^+^CD8αα^+^ IELs isolated from wild-type (WT), *E8*_*I*_^−/−^ and *E8*_*I*_*-*core^−/−^mice. Each dot represents one mouse. Horizontal bars indicate mean values. A one-way ANOVA analysis followed by Tukey's multiple-comparison test was performed for statistical analysis. **(B,D)** The MFI values of WT cells were set as 1. Each dot represents one mouse. Horizontal bars indicate mean values. A one-sample *t*-test was performed for statistical analysis, where the values obtained from each group of the mutant mice were compared to the WT ones. The *p*-values were defined as following: ^**^, *p* < 0.01; ^***^, *p* < 0.001. Data are representative of 5–6 mice **(A,C)** or show the summary of 5–6 mice **(B)** mice, 14–17 mice **(D)** analyzed in 5 **(A,B)** and 16 **(C,D)** independent experiments.

To study the role of E8_I_-core in the regulation of CD8 expression in more detail, we generated E8_I_-core-deficient mice (*E8*_*I*_*-core*^−/−^) using standard gene-targeting approaches (Figure S2, Tables S2, S3). *E8*_*I*_*-*core^−/−^mice displayed no obvious alterations in the percentages and numbers of major T cell subsets in thymus and spleen (Figures S3A–D and data not shown). However, CD8 expression on CD8SP thymocytes as well as CD8^+^ T cells was slightly reduced in the absence of E8_I_-core to a similar degree as observed in *E8*_*I*_^−/−^ mice (Figures 2A,B) (13, 14), indicating that the enhancer activity of E8_I_ in the cytotoxic lineage is largely attributed to the E8_I_-core region. E8_I_ has been shown to control CD8 expression in IEL subsets, particularly in CD8αα homodimers-expressing IELs (13, 14). Similar to the observation made in *E8*_*I*_^−/−^ mice, the deletion of E8_I_-core led to a substantial reduction in the percentage of CD8αα^+^ cells within TCRγδ^+^ IELs, and the residual TCRγδ^+^CD8αα^+^ IELs express CD8αα homodimers at a lower level compared to WT cells (Figures 2C,D). Together, these data indicate that E8_I_-core represents the core enhancer region of E8_I_, and that E8_I_-core regulates CD8 expression in cytotoxic lineage cells.

### Deletion of E8_VI_ Leads to a Reduction in CD8 Expression in Cytotoxic Lineage T Cells

**Figure 3:**
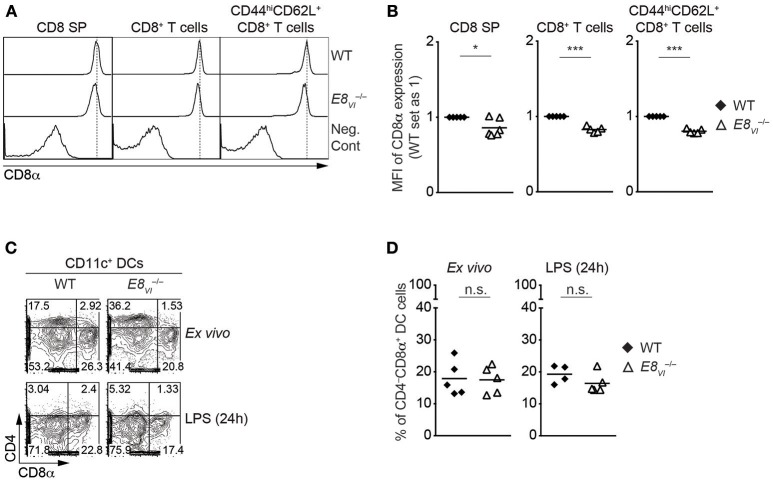
Deletion of *E8*_*VI*_ leads to a reduction in CD8 expression in cytotoxic lineage cells. **(A)** Histograms showing CD8α expression on CD8SP thymocytes, splenic CD8^+^ and CD8^+^CD44^hi^CD62L^+^ T cells isolated from wild-type (WT) and *E8*_*VI*_^−/−^ mice. The gating strategy is shown in Figures S4B,C. Dotted vertical lines indicate the peaks of CD8α expression on WT cells. CD8α expression on WT CD4SP thymocytes (left panel) or CD4^+^ T cells (middle and right panels) is shown as negative staining control. **(B)** Diagrams showing the relative mean fluorescence intensity (MFI) of CD8α expression on CD8SP thymocytes (left), splenic CD8^+^ (middle) and CD8^+^CD44^hi^CD62L^+^ (right) T cells isolated from wild type (WT) and *E8*_*VI*_^−/−^ mice. The MFI values of WT cells were set as 1 for each experiment. Each dot represents one mouse. Horizontal bars indicate mean values. A one-sample *t*-test was performed for statistical analysis, where the values obtained from *E8*_*VI*_^−/−^ mice were compared to the WT ones (i.e., 1). The *p*-values were defined as following: ^*^, *p* < 0.05; ^***^, *p* < 0.001. **(C)** Flow cytometry analysis showing CD8α and CD4 expression on wild-type (WT) and *E8*_*VI*_^−/−^ splenic dendritic cells (DCs), which were either freshly isolated (upper panel) or stimulated with LPS for 24 h (lower panel). The gating strategy for DCs is shown in Figure S4G. Numbers indicate the percentages within the respective regions. Data are representative of 4–5 mice analyzed in 4 independent experiments. **(D)** Diagrams showing the percentage of the CD4^−^CD8α^+^ population within wild-type (WT) and *E8*_*VI*_^−/−^ splenic dendritic cells (DCs), which were either freshly isolated (left) or stimulated with LPS for 24 h (right). Each dot represents one mouse. Horizontal bars indicate mean values. An unpaired Student's *t*-test was performed for statistical analysis. n.s., not significant. Data are representative **(A,C)** or show the summary **(B,D)** of 5–6 mice **(B)** and 4–5 mice **(B)** analyzed in 5 **(A,B)** and 4 **(C,D)** independent experiments.

The analysis of ATACseq peaks in the ImmGen database revealed a similar developmental regulation of chromatin accessibility at E8_I_-core and E8_VI_ (Figure 1), suggesting that E8_VI_ might compensate for loss of E8_I_. Previous transgenic reporter gene expression assays revealed that E8_VI_, which overlaps with ECR-4, is active in mature CD8SP thymocytes and cytotoxic T cells, particularly in CD44^hi^CD62L^+^ effector CD8^+^ T cells, as well as in CD8αα^+^ DCs (23). However, whether E8_VI_ is essential for CD8 expression has not been analyzed by genetic approaches. In order to delete the genomic region harboring E8_VI_ we utilized the CRISPR/Cas9 system. Two guide RNAs complementary to upstream and downstream sequences of E8_VI_ were injected into fertilized eggs together with *Cas9* mRNA (Table S4). The resulting offspring were screened by PCR (Figure S4A) and successful deletion was confirmed by sequencing (Table S5). Mice containing the *E8*_*VI*_-deficient allele were crossed with C57BL/6 mice, and were subsequently intercrossed to generate *E8*_*VI*_^−/−^ mice (Figure S2C). *E8*_*VI*_^−/−^ mice displayed no obvious alteration in the percentage and number of major thymic and splenic T cell subsets compared to littermate control wild-type (WT) mice, indicating that T cell development is largely intact in the absence of E8_VI_ (Figures S4B–D, and data not shown). However, we noticed a mild reduction in CD8 expression levels on E8_VI_-deficient CD8SP thymocytes (Figures 3A,B and Figure S4B), while CD8 expression level on HSA^hi^TCRβ^lo^ DP thymocytes in the absence E8_VI_ was unchanged (Figures S4E,F). A similar mild reduction in CD8 expression levels was also observed in E8_VI_-deficient splenic CD8^+^ T cells (Figures 3A,B), indicating that E8_VI_ contributes to the induction and/or maintenance of CD8 expression in cytotoxic lineage cells. We previously observed a preferential activity of E8_VI_ in the effector/memory CD44^hi^CD62L^+^ subset within the peripheral CD8^+^ T cell compartment (23), therefore we examined CD8 expression on splenic CD8^+^CD44^hi^CD62L^+^ T cells in the absence of E8_VI_. E8_VI_-deficient CD8^+^CD44^hi^CD62L^+^ T cells also showed a reduction in CD8 expression levels, although to a similar degree as observed in total CD8^+^ T cells (Figures 3A,B, and Figure S4C). This indicates that E8_VI_ is not preferentially utilized by effector/memory T cells to drive CD8 expression. Since E8_VI_ is also active in CD8αα^+^ DCs (23), we assessed CD8 expression in splenic *E8*_*VI*_^−/−^ CD11c^+^ DCs (Figures 3C,D and Figure S4G). WT and E8_VI_-deficient DC cells, both *ex vivo* analyzed and after LPS-stimulation, had a similar fraction of the CD4^−^CD8α^+^ subset, suggesting a dispensable role of E8_VI_ for CD8 expression in DCs. Together, these results indicate that E8_VI_ is required for appropriate CD8 expression in cytotoxic lineage T cells and that loss of E8_VI_ cannot be fully compensated by other *Cd8* enhancers.

### E8_I_-Core and E8_VI_ Synergistically Regulate CD8 Expression in Cytotoxic Lineage T Cells

**Figure 4:**
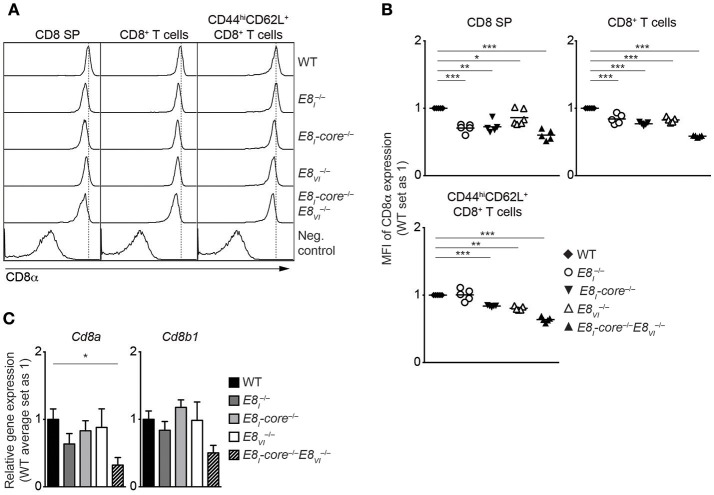
*E8*_*I*_*-*core and *E8*_*VI*_ synergistically regulate CD8 expression in cytotoxic lineage cells. **(A)** Histograms depict CD8α expression on CD8SP thymocytes, splenic CD8^+^ and CD8^+^CD44^hi^CD62L^+^ T cells isolated from wild-type (WT), *E8*_*I*_^−/−^, *E8*_*I*_*-*core^−/−^, *E8*_*VI*_^−/−^ and *E8*_*I*_*-core*^−/−^*E8*_*VI*_^−/−^ mice. The gating strategy is shown in Figures S4B,D. Dotted vertical lines indicate the peaks of CD8α expression on WT cells. CD8α expression on WT CD4SP thymocytes (left panel) or CD4^+^ T cells (middle and right panels) is shown as negative staining controls. **(B)** Diagrams showing the relative mean fluorescence intensity (MFI) of CD8α expression on CD8SP thymocytes (left), splenic CD8^+^ (middle) and CD8^+^CD44^hi^CD62L^+^ (right) T cells isolated from wild type (WT), *E8*_*I*_^−/−^, *E8*_*I*_*-*core^−/−^, *E8*_*VI*_^−/−^, and *E8*_*I*_*-core*^−/−^*E8*_*VI*_^−/−^ mice. Data obtained from the analysis of WT, *E8*_*I*_^−/−^, *E8*_*I*_*-*core^−/−^ and *E8*_*VI*_^−/−^ mice (already shown in Figures 2B, 3B) were included in the respective diagrams. The MFI values of WT cells are set as 1. Each dot represents one mouse. Horizontal bars indicate mean values. A one-sample *t*-test was performed for statistical analysis, where the values obtained from each group of the mutant mice were compared to the WT ones (i.e., 1). Only the comparisons that reached statistically significant levels (i.e., *p* < 0.05) are indicated in the diagrams. **(C)** qRT-PCR analysis showing *Cd8a* (left) and *Cd8b1* (right) gene expression levels (normalized to the *Hprt* gene expression levels) in wild-type (WT), *E8*_*I*_^−/−^, *E8*_*I*_*-*core^−/−^, *E8*_*VI*_^−/−^ and *E8*_*I*_*-core*^−/−^*E8*_*VI*_^−/−^ naive CD44^lo^CD62L^+^CD8^+^ T cells. The average expression levels in WT cells were set as 1. Error bars indicate SEM. A one-way ANOVA analysis followed by Tukey's multiple-comparison test was performed for statistical analysis. **(B,C)** The *p*-values were defined as following: ^*^, *p* < 0.05; ^**^, *p* < 0.01; ^***^, *p* < 0.001. Data are representative **(A)** or show the summary **(B,C)** of 5–6 mice **(A,B)** or 4–5 independent biological samples **(C)** analyzed in 5 **(A,B)** and 5 **(C)** independent experiments.

To investigate potential synergistic and/or redundant activities of E8_I_-core and E8_VI_, we next targeted the E8_VI_ region in *E8*_*I*_*-core*^−/−^ embryos by using the same CRISPR/Cas9 approach as described above, resulting in the generation of E8_I_-core/E8_VI_-doubly deficient mice (*E8*_*I*_*-*core^−/−^*E8*_*VI*_^−/−^) (Figure S2C, Tables S4, S5). These mice were then analyzed and compared to WT, the “original” E8_I_-deficient (${E8}_{I}^{-/-}$), *E8*_*I*_*-*core^−/−^ and *E8*_*VI*_^−/−^ mice. While T cell development is largely intact in *E8*_*I*_*-*core^−/−^*E8*_*VI*_^−/−^ mice (Figures S5A,B and data not shown), the combined deletion of E8_I_-core and E8_VI_ led to a further reduction in CD8 expression levels compared to the individual E8_I_-core and E8_VI_ mutant mice, indicating that E8_I_-core and E8_VI_ synergistically regulate CD8 expression in CD8SP thymocytes (Figures 4A,B). A similar pattern of CD8 downmodulation was also observed in splenic total and effector/memory (CD44^hi^CD62L^+^) CD8^+^ T cell populations in *E8*_*I*_*-*core^−/−^, *E8*_*VI*_^−/−^and *E8*_*I*_*-*core^−/−^*E8*_*VI*_^−/−^ mice, while $E8_{I}^{\;-/-}$ CD8^+^CD44^hi^CD62L^+^ T cells did not show a reduction in CD8 expression compared to WT cells (Figures 4A,B). The CD8 coreceptor on CD8^+^ T cells consists of CD8α and CD8β chains, and CD8β requires CD8α expression for cell surface expression (38). In order to examine whether the downmodulation of CD8 in the mutant CD8^+^ T cells is due to impaired transcription of either *Cd8a* only or of both *Cd8a* and *Cd8b1* we analyzed mRNA expression of these two genes. qRT-PCR analysis revealed reduced expression of *Cd8a* and also a strong tendency of reduced *Cd8b1* expression in naïve *E8*_*I*_*-*core^−/−^*E8*_*VI*_^−/−^ CD8^+^ T cells compared to WT cells (Figure 4C), indicating that the combined deletion of *E8*_*I*_*-*core^−/−^ and of *E8*_*VI*_^−/−^ affects the whole *Cd8ab1* gene complex. Of note, we observed only a tendency of reduced *Cd8a* expression levels in the individual mutant mice based on the mean relative expression levels. This is most likely due to experimental variance when comparing 5 groups that makes it difficult to detect a 20% difference on protein level also on RNA level. Finally, we analyzed CD8 expression in *ex vivo* and LPS-stimulated CD11c^+^ DCs isolated from WT, *E8*_*I*_^−/−^, *E8*_*I*_*-*core^−/−^, *E8*_*VI*_^−/−^, and *E8*_*I*_*-*core^−/−^*E8*_*VI*_^−/−^ mice, and observed no alteration in the proportion of the CD8α^+^CD4^−^ subset in all the mutant mice (Figures S5C,D). Together, these data suggest that E8_I_-core and E8_VI_ synergistically regulate CD8 expression in cytotoxic lineage cells. However, both enhancers are not essential for directing CD8α expression in DCs.

### Synergistic Activity of E8_I_-Core and E8_VI_ Is Required for the Maintenance of CD8 Expression on Activated CD8^+^ T Cells

**Figure 5:**
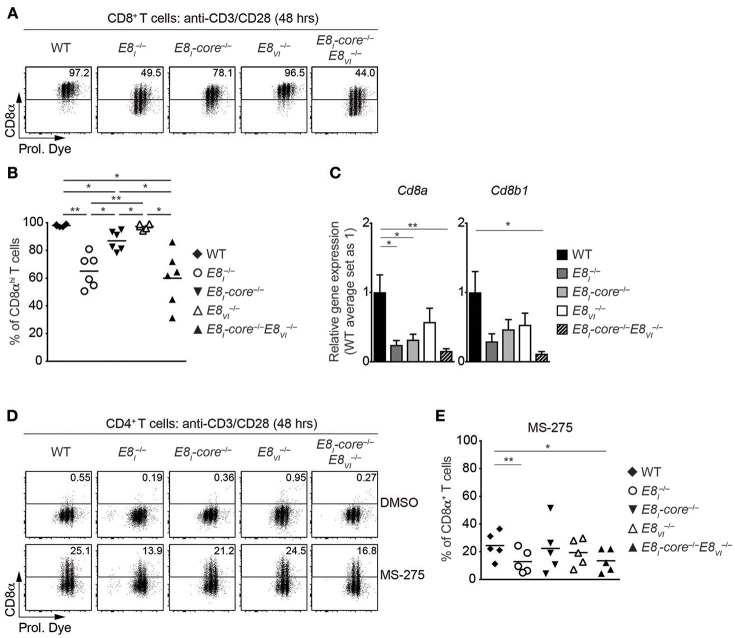
E8_I_-core and E8_VI_ function in activated CD8^+^ and HDAC inhibitor-treated CD4^+^ T cells. **(A)** Flow cytometry analysis showing CD8α expression and Cell proliferation dye (Prol. Dye) dilution on activated CD8^+^ T cells isolated from wild-type (WT), *E8*_*I*_^−/−^, *E8*_*I*_*-*core^−/−^, *E8*_*VI*_^−/−^, and *E8*_*I*_*-*core^−/−^*E8*_*VI*_^−/−^ mice. Naïve CD8^+^ T cells were stimulated with plate-bound anti-CD3 and anti-CD28 antibodies for 48 h. Numbers indicate the percentages of the CD8α^hi^ population. **(B)** Diagrams showing the percentages of CD8α^hi^ population within activated CD8^+^ T cells (48 h after activation), isolated from wild-type (WT), *E8*_*I*_^−/−^, *E8*_*I*_*-*core^−/−^, *E8*_*VI*_^−/−^ and *E8*_*I*_*-core*^−/−^*E8*_*VI*_^−/−^ mice. **(C)** qRT-PCR analysis showing *Cd8a* (left) and *Cd8b1* (right) gene expression levels (normalized to the *Hprt* gene expression levels) in wild-type (WT), *E8*_*I*_^−/−^, *E8*_*I*_*-core*^−/−^, *E8*_*VI*_^−/−^ and *E8*_*I*_*-*core^−/−^*E8*_*VI*_^−/−^ activated CD8^+^ T cells (72 h after activation). The average expression levels in WT cells were set as 1. The summary of 5–6 biologically independent experiments is shown. Error bars indicate SEM. A one-way ANOVA analysis with repeated measures followed by Tukey's multiple-comparison test was performed for statistical analysis. **(D)** Naïve CD4^+^ T cells were stimulated with plate-bound anti-CD3 and anti-CD28 antibodies for 48 h, and were cultured in the presence of DMSO or MS-275 for the last 24 h. Flow cytometry analysis showing CD8α expression and Cell Proliferation Dye (Prol. Dye) dilution in DMSO- (upper panel) and MS-275-treated (lower panel) activated CD4^+^ T cells isolated from wild-type (WT), *E8*_*I*_^−/−^, *E8*_*I*_*-*core^−/−^, *E8*_*VI*_^−/−^, and *E8*_*I*_*-core*^−/−^*E8*_*VI*_^−/−^ mice. Numbers indicate the percentages of CD8α^+^ cells. **(E)** Diagrams showing the percentage of CD8α^+^ cells within MS-275-treated activated CD4^+^ T cells isolated from wild-type (WT), *E8*_*I*_^−/−^, *E8*_*I*_*-core*^−/−^, *E8*_*VI*_^−/−^ and *E8*_*I*_*-*core^−/−^*E8*_*VI*_^−/−^ mice. **(B,E)** Each dot represents one mouse. Horizontal bars indicate mean values. A one-way ANOVA analysis with repeated measures followed by Tukey's multiple-comparison test was performed for statistical analysis. **(B,C,E)** The *p*-values were defined as following: ^*^, *p* < 0.05; ^**^, *p* < 0.01; ^***^, *p* < 0.001. Data are representative **(A,D)** or show the summary **(B,C,E)** of 6 mice **(A,B)**, 5–6 samples **(C)** and 5 mice **(D,E)** analyzed in 6 **(A,B)**, 5–6 **(C)**, and 4 **(D,E)** independent experiments.

Our previous study has demonstrated that E8_I_ is required for the maintenance of CD8 expression on CD8^+^ T cells upon activation (18). We therefore examined the individual and combinatorial roles of E8_I_-core and E8_VI_ in activated CD8^+^ T cells. Naïve CD8^+^ T cells from WT, $E8_{I}^{\;-/-}$, *E8*_*I*_*-core*^−/−^, $E8_{VI}^{\;-/-}$, and *E8*_*I*_*-*core^−/−^$E8_{VI}^{\;-/-}$ mice were stimulated with anti-CD3/CD28, and were analyzed for CD8 expression 48 h after activation. The mutant CD8^+^ T cells displayed comparable proliferative capacity to WT cells (Figure 5A). Consistent with our previous study (18), almost half of $E8_{I}^{\;-/-}$ CD8^+^ T cells downmodulate CD8 expression 48 h after activation (Figures 5A,B). Interestingly, *E8*_*I*_*-*core^−/−^ cells displayed a milder CD8 downmodulation compared to $E8_{I}^{\;-/-}$ cells, suggesting that another region within E8_I_ contributes to the maintenance of CD8 expression. In addition, while *E8*_*VI*_^−/−^ CD8^+^ T cells maintained CD8 expression at a similar level as WT cells (albeit a tendency toward a lower proportion of CD8^hi^ cells was observed), the deletion of both E8_I_-core and E8_VI_ led to enhanced CD8 downmodulation, compared to the single mutant cells. qRT-PCR analysis showed that the combined deletion of *E8*_*I*_*-*core^−/−^ and *E8*_*VI*_^−/−^ led to a reduced expression of both *Cd8a* and *Cd8b1* in activated CD8^+^ T cells (Figure 5C). Together, these data indicate that the maintenance of CD8 expression is regulated by E8_I_-core and an additional *cis*-region within E8_I_, and that the synergistic activity of E8_I_-core and E8_VI_ plays an important role for the maintenance of CD8 expression upon activation.

### E8_I_-Core and E8_VI_ Contribute to Class I HDAC Inhibitor Treatment-Induced CD8 Expression in CD4^+^ T Cells

In addition to its role in regulating CD8 expression in cytotoxic T cells, E8_I_ displays also activity in helper lineage T cells. We have previously demonstrated that HDAC1 and HDAC2 are required for the maintenance of the lineage integrity of CD4^+^ T cells, and that treatment with class I HDAC inhibitor MS-275 of activated CD4^+^ T cells leads to the induction of CD8α and CD8β expression in an E8_I_-dependent manner (21). In order to test the role of E8_I_-core and E8_VI_ for CD8 induction in CD4^+^ T cells, we activated *E8*_*I*_^−/−^, *E8*_*I*_*-*core^−/−^, *E8*_*VI*_^−/−^ and *E8*_*I*_*-*core^−/−^$E8_{VI}^{\;-/-}$ CD4^+^ T cells in the presence of MS-275, and analyzed CD8 expression (Figures 5D,E). As observed previously, E8_I_-deficient CD4^+^ T cells displayed an impaired upregulation of CD8 compared to WT cells. While *E8*_*I*_*-*core^−/−^ and $E8_{VI}^{\;-/-}$ CD4^+^ T cells upregulated CD8 expression to a similar degree as WT cells, the combined deletion of E8_I_-core and E8_VI_ led to a reduction in the proportion of CD4^+^ T cells that expressed CD8. This indicates a synergistic activity of E8_I_-core and E8_VI_ in HDAC inhibitor-mediated CD8 induction in CD4^+^ T cells.

### E8_I_-Core and E8_VI_ Regulate CD8 Expression in IEL Subsets

**Figure 6:**
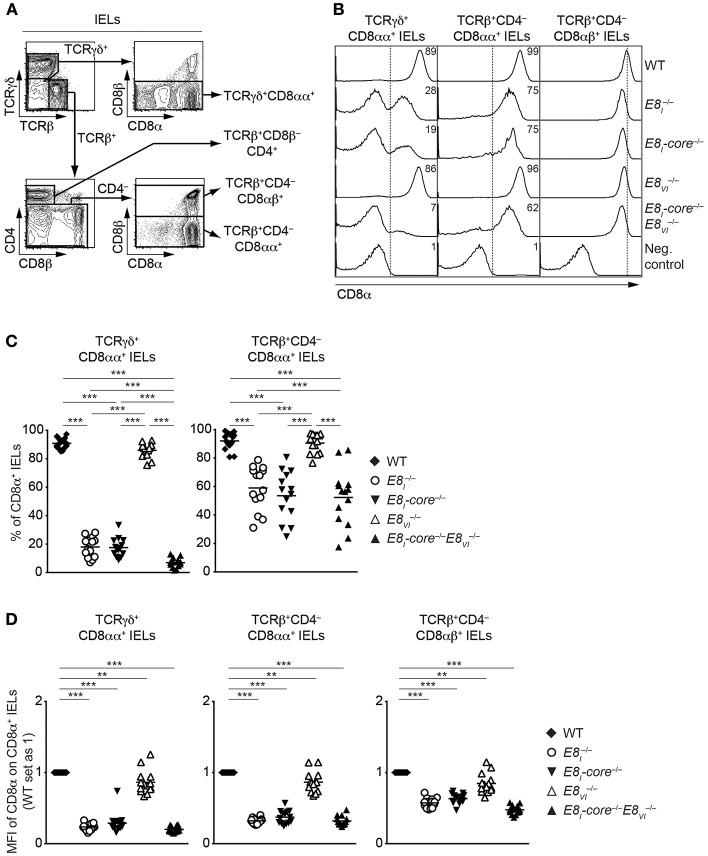
CD8αα expression on IELs is predominantly regulated by *E8*_*I*_*-core*. **(A)** Representative gating strategy for the analysis of CD8α expression on TCRγδ^+^CD8αα^+^, TCRβ^+^CD8β^−^CD4^+^, TCRβCD4^−^CD8αα^+^, TCRβ^+^CD4^−^CD8αβ^+^, and IELs. **(B)** Histograms showing CD8α expression on TCRγδ^+^CD8αα^+^, TCRβ^+^CD4^−^CD8αα^+^, and TCRβ^+^CD4^−^CD8αβ^+^ IELs isolated from wild-type (WT), *E8*_*I*_^−/−^, *E8*_*I*_*-*core^−/−^, *E8*_*VI*_^−/−^ and *E8*_*I*_*-*core^−/−^*E8*_*VI*_^−/−^ mice. CD8α expression on WT CD19^+^ B cells is shown as a negative staining control. Dotted lines indicate either gating region for the CD8α^+^ population (TCRγδ^+^CD8αα^+^ and TCRβ^+^CD4^−^CD8αα^+^ IELs) or the peak of CD8α expression on WT cells (TCRβ^+^CD4^−^CD8αβ^+^ IELs). Numbers indicate the percentages of CD8α^+^ cells. **(C)** Diagrams showing the percentage of CD8α^+^ cells within TCRγδ^+^CD8αα^+^, and TCRβ^+^CD4^−^CD8αα^+^ IELs isolated from wild-type (WT), *E8*_*I*_^−/−^, *E8*_*I*_*-*core^−/−^, *E8*_*VI*_^−/−^ and *E8*_*I*_*-*core^−/−^*E8*_*VI*_^−/−^ mice. Data obtained from the analysis of WT, *E8*_*I*_^−/−^, *E8*_*I*_*-*core^−/−^ TCRγδ^+^CD8αα^+^ IELs (as shown in Figure 2D, left panel) were included in the corresponding diagram. Each dot represents one mouse. Horizontal bars indicate mean values. A one-way ANOVA analysis followed by Tukey's multiple-comparison test was performed for statistical analysis. **(D)** Diagrams showing the relative mean fluorescence intensity (MFI) of CD8α expression within the CD8α^+^ population of TCRγδ^+^CD8αα^+^, TCRβ^+^CD4^−^CD8αα^+^, and TCRβ^+^CD4^−^CD8αβ^+^ IELs isolated from wild type (WT), *E8*_*I*_^−/−^, *E8*_*I*_*-*core^−/−^, *E8*_*VI*_^−/−^ and *E8*_*I*_*-*core^−/−^*E8*_*VI*_^−/−^ mice. The MFI values of WT cells are set as 1. Data obtained from the analysis of WT, *E8*_*I*_^−/−^, *E8*_*I*_*-core*^−/−^ TCRγδ^+^CD8αα^+^ IELs (shown in Figure 2D, right panel) were included in the corresponding diagrams. Each dot represents one mouse. Horizontal bars indicate mean values. A one-sample *t*-test was performed for statistical analysis, where the values obtained from each group of the mutant mice were compared to the WT ones (i.e., 1). **(C,D)** The *p*-values were defined as following: ^**^, *p* < 0.01; ^***^, *p* < 0.001. Data are representative **(B)** or show the summary **(C,D)** of 14–17 mice **(B,C,D)** analyzed in 16 **(B,C,D)** independent experiments.

The IEL population consists of both γδ and αβ T cell lineages. Whereas, TCRγδ^+^ IELs predominantly express CD8αα homodimers, TCRαβ^+^CD8α^+^ IELs express either CD8αα homodimers or CD8αβ heterodimers (39). Since E8_I_ controls CD8α expression in IELs (13, 14), we performed a comprehensive analysis of CD8 expression on these IEL subsets isolated from WT, *E8*_*I*_^−/−^, *E8*_*I*_*-*core^−/−^, *E8*_*VI*_^−/−^ and *E8*_*I*_*-*core^−/−^$E8_{VI}^{\;-/-}$ mice (Figure 6A). Unlike E8_I_- or E8_I_-core-deficient TCRγδ^+^CD8αα^+^ IELs (Figures 2C,D, 6B, left column, and Figure 6C), *E8*_*VI*_^−/−^ TCRγδ^+^CD8αα^+^ IELs showed no alterations in the proportion of CD8-expressing cells (Figure 6B, left column, and Figure 6C), although a mild reduction in CD8α expression levels was observed (Figure 6D). In contrast, the combined deletion of both E8_I_-core and E8_VI_ led to an almost complete loss of CD8 expression (Figure 6B, left column, and Figures 6C,D), suggesting that these two enhancers control CD8αα expression in TCRγδ^+^ IELs synergistically. In the TCRβ^+^CD4^−^CD8αα^+^ IEL population, *E8*_*I*_*-*core^−/−^ mice displayed a similar reduction of CD8 expression levels as observed in *E8*_*I*_^−/−^ cells (Figure 6B, middle column, and Figures 6C,D), indicating that the E8_I_ enhancer activity directing CD8αα expression in TCRγδ^+^ and TCRαβ^+^ IELs resides predominantly in the E8_I_-core region. In $E8_{VI}^{\;-/-}$ mice, there was no reduction of the percentage of TCRαβ^+^ CD8αα-expressing IELs (Figure 6B, middle column, and Figure 6C), although a mild reduction in CD8α expression levels was observed in the absence of E8_VI_ (Figure 6D). However, unlike in *E8*_*I*_*-*core^−/−^*E8*_*VI*_^**−/−**^ TCRγδ^+^CD8αα^+^ IELs, *E8*_*I*_*-*core^−/−^*E8*_*VI*_^**−/−**^ TCRβ^+^CD4^−^CD8αα^+^ IELs displayed no further reduction in CD8αα expression in comparison to *E8*_*I*_*-*core^−/−^ cells (Figure 6B, middle column, and Figures 6C,D), suggesting that E8_VI_ plays only a minor role in the regulation of CD8 expression in TCRβ^+^CD4^−^CD8αα^+^ IELs. Finally, we investigated CD8αβ expression on TCRβ^+^CD4^−^CD8αβ^+^ IELs in the various mutant mice (Figure 6B, right column, and Figure 6D). In the absence of either E8_I_-core or E8_VI_, the CD8αβ expression level was reduced, and CD8αβ levels were further reduced upon combined loss of both enhancers (Figure 6D), which is reminiscent of the expression pattern on peripheral CD8^+^ T cells (Figures 4A,B). Together, these analyses revealed that CD8αα expression in IELs (particularly on TCRγδ^+^CD8αα^+^ IELs) largely depends on E8_I_-core enhancer activity, whereas E8_VI_ provides a minor contribution to CD8α expression.

### E8_I_-Core Is Required for the Acquisition of Cytotoxic Features of TCRαβ^+^ CD4^+^ IELs

**Figure 7:**
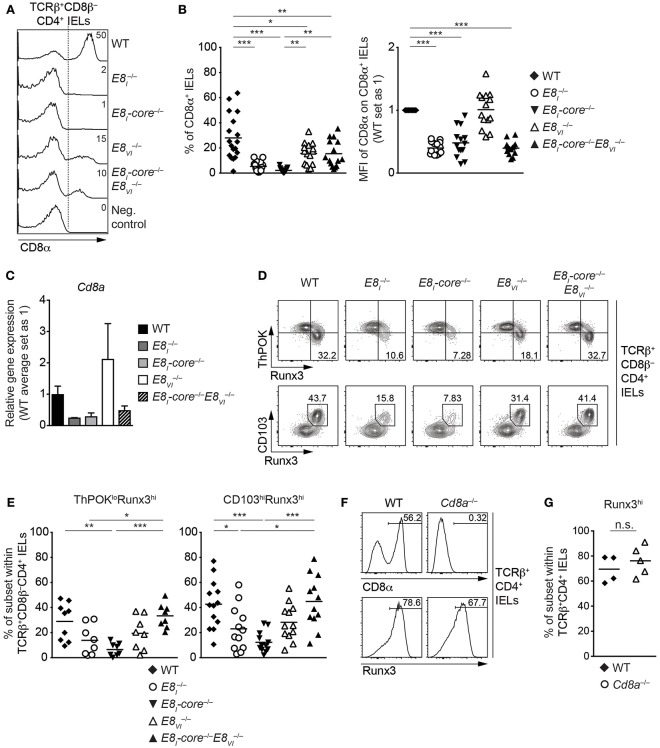
*E8*_*I*_*-core* is required for the generation of CD4^+^ CTLs in the small intestine. **(A)** Histograms showing CD8α expression on TCRβ^+^CD8β^−^CD4^+^ IELs isolated from wild-type (WT), *E8*_*I*_^−/−^, *E8*_*I*_*-*core^−/−^, *E8*_*VI*_^−/−^ and *E8*_*I*_*-core*^−/−^*E8*_*VI*_^−/−^ mice. The gating strategy is shown in Figure 6A. CD8α expression on WT CD19^+^ B cells is shown as a negative control for the staining. Dotted lines and numbers indicate gating region for the CD8α^+^ population and the percentages of the CD8α^+^ population, respectively. **(B)** Diagrams showing the percentage of the CD8α^+^ population (left) and the relative mean fluorescence intensity (MFI) of CD8α expression on the CD8α^+^ population (right) within TCRβ^+^CD8β^−^CD4^+^ IELs isolated from wild type (WT), *E8*_*I*_^−/−^, *E8*_*I*_*-core*^−/−^, *E8*_*VI*_^−/−^ and *E8*_*I*_*-core*^−/−^*E8*_*VI*_^−/−^ mice. Each dot represents one mouse. Horizontal bars indicate mean values. The MFI values of WT cells are set as 1. A one-way ANOVA analysis followed by Tukey's multiple-comparison test (left) or a one-sample *t*-test, where the values obtained from each group of the mutant mice were compared to the WT ones (i.e., 1) (right), was performed. **(C)** qRT-PCR analysis showing *Cd8a* gene expression levels (normalized to the *Hprt* gene expression levels) in sorted TCRβ^+^CD8β^−^CD4^+^ IELs from wild-type (WT), *E8*_*I*_^−/−^, *E8*_*I*_*-*core^−/−^, *E8*_*VI*_^−/−^, and *E8*_*I*_*-core*^−/−^*E8*_*VI*_^−/−^ mice. The average expression levels in WT cells were set as 1. Error bars indicate SEM. A one-way ANOVA analysis followed by Tukey's multiple-comparison test was performed for statistical analysis. **(D)** Flow cytometry analysis showing Runx3 and ThPOK (upper panel) and Runx3 and CD103 (lower panel) expression on TCRβ^+^CD8β^−^CD4^+^ IELs isolated from wild type (WT), *E8*_*I*_^−/−^, *E8*_*I*_*-core*^−/−^, *E8*_*VI*_^−/−^ and *E8*_*I*_*-core*^−/−^*E8*_*VI*_^−/−^ mice. Numbers indicate the percentages of ThPOK^lo^Runx3^hi^ subsets (upper panel) and CD103^hi^Runx3^hi^ subset (lower panel). **(E)** Diagrams showing the percentages of the ThPOK^lo^Runx3^hi^ (left panel) and CD103^hi^Runx3^hi^ (right panel) subsets within TCRβ^+^CD8β^−^CD4^+^ IELs isolated from wild type (WT), *E8*_*I*_^−/−^, *E8*_*I*_*-core*^−/−^, *E8*_*VI*_^−/−^ and *E8*_*I*_*-core*^−/−^*E8*_*VI*_^−/−^ mice. Each dot represents one mouse. Horizontal bars indicate mean values. A one-way ANOVA analysis followed by Tukey's multiple-comparison test was performed for statistical analysis. **(B,E)** The *p*-values were defined as following: ^*^, *p* < 0.05; ^**^, *p* < 0.01; ^***^, *p* < 0.001. **(F)** Histograms showing CD8α (upper panel) and Runx3 (lower panel) expression on TCRβ^+^CD8β^−^CD4^+^ IELs isolated from wild type (WT) and *Cd8a*^−/−^ mice. Numbers indicate the percentages of respective regions. **(G)** Diagrams showing the percentages of the Runx3^hi^ subset within TCRβ^+^CD8β^−^CD4^+^ IELs isolated from wild type (WT) and *Cd8a*^−/−^ mice. Each dot represents one mouse. Horizontal bars indicate mean values. An unpaired Student's *t*-test was performed for statistical analysis. n.s., not significant. Data are representative **(A,D,F)** or show the summary of 15-18 mice **(A,B)**, 3–4 independent biological samples **(C)**, 8–13 mice **(D,E)** and 4–5 mice **(F,G)** analyzed in 16 **(A,B)**, 2 **(C)** 8–12 **(D,E)**, and 2 **(F,G)** independent experiments.

It has been shown that a fraction of mature TCRαβ^+^CD4^+^ T cells acquires cytotoxic features in the intestine. The generation of these CD4 CTLs from CD4^+^ T cells is controlled by a transcriptional reprogramming of ThPOK and Runx3 expression that leads to the downmodulation of ThPOK and the upregulation of Runx3 (19, 20). Since E8_I_ is required for the induction of CD8αα in CD4 CTLs (20), we investigated CD8αα expression in TCRβ^+^CD8β^−^CD4^+^ IELs isolated from WT, *E8*_*I*_^−/−^, *E8*_*I*_*-*core^−/−^, *E8*_*VI*_^−/−^ and *E8*_*I*_*-*core^−/−^*E8*_*VI*_^**−/−**^ mice (Figures 7A,B). Similar to the phenotype observed in *E8*_*I*_^−/−^ mice, the deletion of E8_I_-core led to an almost complete loss of CD8αα-expressing subsets within TCRβ^+^CD8β^−^CD4^+^ IELs and the few cells that still expressed CD8α displayed reduced CD8α expression levels (Figures 7A,B). We also observed reduced levels of *Cd8a* gene expression in total $E8_{I}^{\;-/-}$ and *E8*_*I*_*-*core^−/−^ TCRβ^+^CD8β^−^CD4^+^ IELs (*p* = 0.0563 and *p* = 0.0759, respectively; based on an unpaired two-tailed Student's *t*-test) (Figure 7C). $E8_{VI}^{\;-/-}$ TCRβ^+^CD8β^−^CD4^+^ IELs displayed an approx. 1.8-fold reduction in the proportion of CD8αα^+^ cells in comparison to WT cells (Figures 7A,B). This suggests that E8_VI_ contributes to the induction of CD8αα expression in TCRβ^+^ CD4^+^ IELs, although the CD8α^+^ cells expressed CD8α at the same level as WT cells (Figures 7A,B) and there was also no detectable difference in *Cd8a* gene expression levels in TCRβ^+^CD8β^−^CD4^+^ IELs (Figure 7C). Of note, the combined deletion of both E8_I_-core and E8_VI_ resulted in the appearance of CD8αα-expressing TCRβ^+^CD8β^−^CD4^+^ IEL subsets with a similar frequency as observed in $E8_{VI}^{\;-/-}$ IELs, although CD8αα expression levels in those cells that express CD8α remained at similar low levels as observed in *E8*_*I*_*-core*^−/−^ IELs (Figures 7A,B). In agreement with the low CD8α protein expression levels, we also observed a tendency that *Cd8a* gene expression is reduced in TCRβ^+^CD8β^−^CD4^+^ IELs (Figure 7C).

The increase in the percentage of CD8αα-expressing TCRβ^+^CD8β^−^CD4^+^ IELs in *E8*_*I*_*-*core^−/−^*E8*_*VI*_^**−/−**^ mice in comparison to *E8*_*I*_*-*core^−/−^ mice was unexpected, since E8_I_-core and E8_VI_ showed synergistic activities in conventional CD8^+^ T cells (Figure 4). Since CD8α expression is a marker for the appearance of CD4 CTLs, we next analyzed the expression of CD103, ThPOK, and Runx3 in the various *Cd8* enhancer mutant TCRβ^+^CD8β^−^CD4^+^ IELs (Figures 7D,E). The deletion of E8_I_-core led to a reduction in the percentage of ThPOK^lo^Runx3^hi^ cells in comparison to WT cells (Figure 7D, upper panel, and Figure 7E), In addition, the frequency of CD103^hi^Runx3^hi^ cells was reduced in *E8*_*I*_^−/−^ and *E8*_*I*_*-*core^−/−^ TCRβ^+^CD8β^−^CD4^+^ IELs (Figure 7D, lower panel, and Figure 7E), indicating that not only CD8α expression but also the generation of CD4 CTLs is impaired in the absence of E8_I_-core. In contrast, the deletion of E8_VI_ did not alter the fraction of ThPOK^lo^Runx3^hi^ or CD103^hi^Runx3^hi^ cells. Interestingly, the combined deletion of E8_I_-core and E8_VI_ reverted the impaired CD4 CTL differentiation caused by loss of E8_I_-core. To test whether the inhibition of CD4 CTL differentiation was caused by impaired CD8αα expression in the absence of the *Cd8* enhancers, we assessed the appearance of Runx3^hi^ cells as a marker for CD4 CTLs in TCRβ^+^CD4^+^ IELs isolated from *Cd8a*^−/−^ mice (Figures 7F,G). Strikingly, *Cd8a*^−/−^ TCRβ^+^CD4^+^ IELs contained similar percentages of Runx3^hi^ cells compared to WT cells, demonstrating that the induction of CD4 CTLs is not dependent on CD8αα expression. Together, these results suggest that loss of E8_I_-core impairs not only the expression of CD8α but also the differentiation of intestinal CD4 CTLs in a CD8α-independent manner, and that this phenotype is converted upon additional loss of E8_VI_.

## Discussion

**Figure 8:**
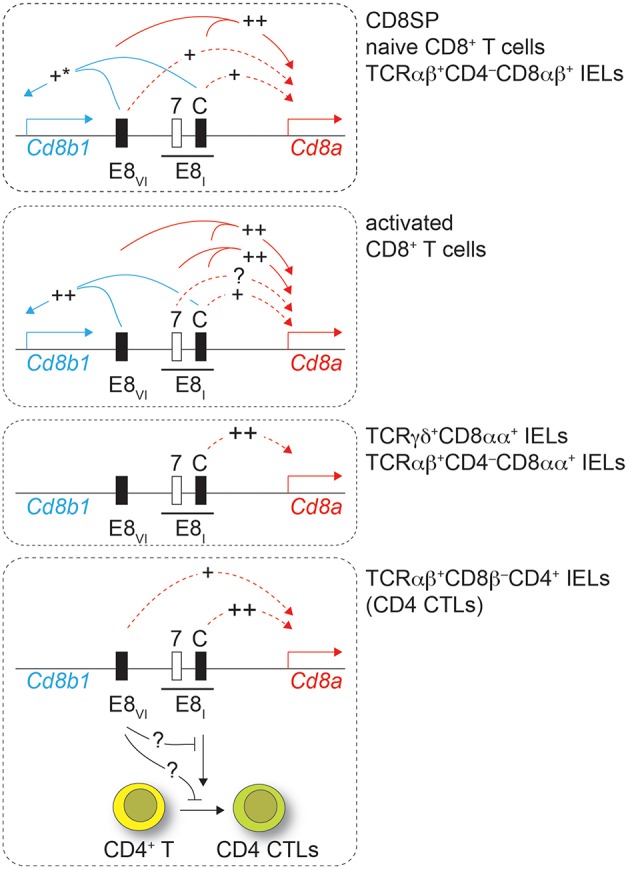
Working model. Drawings depict the *Cd8ab1* gene complex (not in scale) in various T cell subsets as depicted on the right. For simplicity, only E8_I_ and E8_VI_ are shown. The black bar above E8_I_ indicates the 7.6 kb genomic region containing the core region of E8_I_ (C) and ECR-7 (7). Solid lines indicate synergy of E8_I_-core and E8_VI_ as revealed by combined deletion of the respective regions, while dotted lines show the effect of the individual enhancer regions. “++” indicates a strong impact on *Cd8* gene expression, while “+” indicates a moderate impact. “+^*^” in the upper panel indicates a tendency that did not reach statistical significance. Lowest panel: loss of E8_I_ affects also the differentiation of CD4^+^ T cell into intestinal CD4 CTLs, which is partially reverted upon additional loss of E8_VI_. See text for more details.

The expression of CD8 is regulated by a complex regulatory network formed by at least 6 developmental stage and lineage-specific *Cd8* enhancers (8, 9, 23). Among those, the mature enhancer E8_I_, initially identified on a 7.6 kb genomic fragment, is required for CD8α expression in IELs as well as for the maintenance of CD8α expression in activated CD8^+^ T cells (13, 18). In this study we first dissected the activity of a 544 bp genomic region within E8_I_ that becomes accessible only in mature CD8 lineage T cells, as identified by searching the ImmGen ATAC-seq database (22). Results from transgenic reporter gene expression assays strongly indicated that this region represents the core enhancer region of E8_I_. Furthermore, using genetic loss of function approaches, we demonstrated an essential role for E8_I_-core in driving the expression of CD8α in IELs, while E8_I_-core contributes to the maintenance of CD8 expression in activated CD8^+^ T cells to a lesser extent in comparison to the full-length E8_I_. This suggests that beside E8_I_-core other regions within the 7.6kb E8_I_ enhancer are required for the maintenance of CD8 expression in activated CD8^+^ T cells (Figure 8). Interestingly, the ATAC-seq ImmGen database reveals another open chromatin region upstream of the E8_I_-core region within E8_I_, which overlaps with the previously identified ECR-7 (Figure 1) (23). This open chromatin region within E8_I_ is, in addition to DP thymocytes, detected in cytotoxic lineage cells. Thus, ECR-7 might act as an enhancer that maintains CD8 expression in naïve and/or activated cytotoxic T cells and that potentially controls CD8 expression in synergy with E8_I_-core. Previous transgenic reporter gene expression assays revealed only a marginal enhancer activity of ECR-7 in cytotoxic T cells (11), however its activity upon activation of cytotoxic T cells has not been investigated. It would be therefore interesting to further elucidate the role of ECR-7 by targeting ECR-7 alone and in combination with E8_I_-core.

Another interesting aspect with respect to E8_I_ function addresses the activation of E8_I_ during IEL differentiation. TCRαβ^+^CD8αα^+^ IELs, in which E8_I_ directs expression, develop from TCRβ^+^CD5^+^ DN thymocytes progenitors (40). In order to test whether E8_I_ is already active in the precursor population (despite the lack of CD8α expression), we took advantage of *E8*_*I*_-Cre reporter mice that have been crossed on a Rosa26-stop-YFP reporter allele (*E8*_*I*_^RosaYfp^) (25). In these mice, the expression of Cre is driven by a 1.6 kb genomic subfragment of E8_I_, which includes also E8_I_-core, and that has the same enhancer activity as the initially described 7.6 kb E8_I_ enhancer (13). While TCRβ^+^CD8αα^+^ IELs in *E8*_*I*_^RosaYfp^ mice expressed YFP, there were no YFP^+^ cells within the thymic IEL precursors (Figure S6). This indicates that E8_I_ is not active in thymic TCRαβ^+^ IEL precursor cells and that E8_I_ must be activated at a later stage of TCRαβ^+^CD8αα^+^ IEL differentiation.

In this study, we also characterized the *Cd8* enhancer E8_VI_ by using CRISPR/Cas9-mediated gene targeting approaches. In line with our previous transgenic reporter gene expression study that revealed a cytotoxic lineage-specific activity of E8_VI_ (23), the deletion of E8_VI_ led to a reduction in CD8 expression levels in cytotoxic lineage cells. The observed CD8 expression phenotype in the absence of E8_VI_ was rather mild, in part due to the compensatory activity of E8_I_-core, since the combined deletion of E8_VI_ and E8_I_-core resulted in a stronger downregulation of CD8 in the cytotoxic lineage compared to the individual deletion of either E8_I_ or E8_VI_. However, *E8*_*I*_*-core*^−/−^*E8*_*VI*_^−/−^ cytotoxic lineage cells still expressed CD8 approximately at half the levels observed in WT cells, suggesting that other known/unknown *Cd8* enhancers are active in naïve CD8^+^ T cells in the absence of E8_I_ and E8_VI_. Candidate enhancer region(s) might be ECR-7, as discussed above, or E8_II_, since E8_II_ is active in mature CD8^+^ T cells (13). We previously demonstrated that loss of both E8_I_ and E8_II_ leads to variegated expression of CD8 expression in DP thymocytes, leading to the development of CD8-negative DP cells. Those E8_I_,E8_II_-doubly-deficient DP cells that express CD8 have the potential to develop into CD8^+^ T cells, however mature cytotoxic lineage T cells in the absence of E8_I_ and E8_II_ display only ~70% of the CD8 levels in comparison to WT CD8^+^ T cells (34). This demonstrates a role for E8_II_ in mature CD8^+^ T cells. Targeting of E8_II_ or ECR-7 in mice that lack E8_I_-core and E8_VI_ is required to address this issue in more detail. Of note, deletion of E8_VI_ did not affect CD8α expression in TCRγδ^+^ IELs. This is consistent with the observation that the chromatin region surrounding E8_VI_ is not open TCRγδ^+^ IELs as revealed by ATAC-seq (32) (Figure S7).

E8_VI_ was the first *Cd8* enhancer described to direct expression in CD8αα^+^ DCs (23). However, our current study showed that *E8*_*VI*_^−/−^ CD8αα^+^ DCs displayed normal CD8 expression. It is likely that this is due to a compensatory activity of other enhancers. Our results further revealed that E8_I_-core did not compensate for loss of E8_VI_ in DCs, indicating that other enhancers might compensate. Of note, based on the ImmGen ATAC-seq database there is no prominent open chromatin region detectable in the *Cd8ab1* gene complex in CD8αα^+^ DCs, except for a region around the *Cd8a* promoter (Figure S7). This might indicate a differential regulatory mechanism of CD8α expression in DCs compared to CD8^+^ T cells. One might speculate that a CD8αα^+^ DCs precursor requires the activity of known/unknown enhancer(s) during a certain developmental window for the establishment of CD8α expression, and that mature CD8αα^+^ DCs maintain CD8α expression in an enhancer-independent manner, possibly through epigenetic mechanisms. Further studies including ATAC-seq experiments in DC precursors are required to elucidate the underlying mechanisms for *Cd8a* gene expression in DCs.

Previous studies indicated an unexpected role for *Cd8* enhancer E8_I_ in CD4 lineage T cells. HDAC1 and HDAC2 control the lineage integrity of helper T cells. HDAC1/HDAC2-doubly-deficient CD4^+^ T cells or WT CD4^+^ T cells treated with the HDAC inhibitor MS-275 upregulate cytotoxic features, including the expression of CD8, which is dependent on *Cd8* enhancer E8_I_ (21). While we confirmed the role of E8_I_ in these CD4 CTLs in this study, loss of E8_I_-core did not affect the upregulation of CD8 in MS-275-treated CD4^+^ T cells, indicating that another *cis*-region, perhaps ECR-7, within E8_I_ is sufficient to induce CD8 in CD4 CTLs. Similarly, MS-275-treated E8_VI_-deficient CD4^+^ T cells upregulated CD8, indicating that E8_VI_ is not essential in CD4 lineage T cells for the induction of CD8. However, the regulatory interactions and compensatory pathways among *Cd8* enhancers are more complex in CD4^+^ T cells, since the combined deletion of E8_I_-core and E8_VI_ led to a reduction in the proportion of MS-275-treated CD4^+^ T cells that upregulated CD8. This indicates a synergistic activity of E8_I_-core and E8_VI_ in MS-275-mediated CD8 induction on CD4^+^ T cells. Moreover, these data indicate that at least three *cis*-regions contribute to the upregulation of CD8 expression in CD4^+^ T cells, two within E8_I_ and one within E8_VI_, and that as long as two of these three regions are present CD8 is upregulated (Figure 8).

Finally, our study also revealed that E8_I_ not only directs the expression of CD8α during the differentiation of CD4^+^ T cells into CTLs, but also that E8_I_ has an important function during the generation of CD4 CTLs. This conclusion is based on the observation that TCRβ^+^CD8β^−^CD4^+^ IELs contained a reduced population into ThPOK^lo^Runx3^hi^ CD4^+^ CTLs in the absence of E8_I_-core (and to a lesser extent also in the absence of E8_I_). Since we observed ThPOK^lo^Runx3^hi^ CD4 CTLs within TCRβ^+^CD8β^−^CD4^+^ IELs even in the absence of the *Cd8a* gene, the role of E8_I_ in the differentiation of CD4 CTLs is not directly linked to its enhancer function for CD8 expression. This finding is in line with our recent study showing intact CD4 CTL generation in mice with a severe reduction of CD8αα expression levels in TCRβ^+^CD8β^−^CD4^+^ IEL subsets due to the deletion of introns at the *Cd8a* locus (*Cd8a*Δ*int*/Δ*int*) (24). It has been shown that the *Cd8ab1* gene complex can physically interact with the *Cd4* gene locus and that *Cd4 cis*-elements influence *Cd8* expression. This interaction is mediated in part by E8_I_ and by Runx3, which binds to E8_I_, while ThPOK antagonized the association of the *Cd4* and *Cd8ab1* gene loci (41). It is therefore tempting to speculate that a similar mechanism might control CD4 CTL generation. A gene locus essential for CD4 CTL differentiation might require an E8_I_-mediated interaction with the *Cd8ab1* loci for activation, thereby also ensuring co-regulation of *Cd8a* gene expression with the induction of intestinal CD4 CTLs. Of note, the E8_I_-mediated association might be antagonized by E8_VI_, since CD4 CTL generation is restored in E8_I_,E8_VI_-doubly-deficient CD4^+^ T cells. Further studies that include a transcriptome analysis as well as an analysis of the nuclear organization of the *Cd8ab1* gene complex in intestinal CD4^+^ T cells and in CD4 CTLs are required to address the mechanism of how E8_I_ controls the generation of CD4 CTLs.

Taken together, our study demonstrated a complex utilization and interplay of *Cd8* enhancers in cytotoxic T cells and in intestinal IELs. Moreover, we revealed that E8_I_-core controls the generation of intestinal CD4 CTLs by a mechanism independent of its enhancer function for CD8 expression.

## Data Availability

The datasets generated for this study are available on request to the corresponding author.

## Author Contributions

AFG, TP, PH, MA, CT, and MO performed experiments and analyzed the data; SM and IT generated *E8I-core*^−/−^, *E8VI*^−/−^ and *E8I-core*^−/−^*E8VI*^−/−^ mice and analyzed *Cd8a*^−/−^ mice; WE designed the research and wrote the manuscript; SS designed the research, performed experiments, analyzed the data and wrote the manuscript.

### Conflict of Interest Statement

The authors declare that the research was conducted in the absence of any commercial or financial relationships that could be construed as a potential conflict of interest.
