# Supplementary material for: Differential Requirement of Cd8 Enhancers E8I and E8VI in Cytotoxic Lineage T Cells and in Intestinal Intraepithelial Lymphocytes
Source: Front Immunol. 2019 Mar 11;10:409. doi: 10.3389/fimmu.2019.00409 (PMC6421288; doi:10.3389/fimmu.2019.00409)
Supplement: Supplementary file 1 [file Data_Sheet_1.pdf]

Supplementary Material

Differential requirement of *Cd8* enhancers $E8_I$ and $E8_{VI}$ in cytotoxic lineage T cells and in intestinal intraepithelial lymphocytes.

Alexandra Franziska Gülich[1], Teresa Preglej[1,5], Patricia Hamminger[1,5], Marlis Alteneder[1], Caroline Tizian[1,3], Maria Jonah Orola[1,4], Sawako Muroi[2], Ichiro Taniuchi[2], Wilfried Ellmeier[1*], Shinya Sakaguchi[1*]

[1]Division of Immunobiology, Institute of Immunology, Center for Pathophysiology, Infectiology and Immunology, Medical University of Vienna, Vienna, Austria.

[2]Laboratory for Transcriptional Regulation, RIKEN Center for Integrative Medical Sciences (IMS), Yokohama, Japan.

[3]Present address: Institute of Microbiology, Infectious Diseases and Immunology, Charité - University Medicine Berlin, Berlin, Germany

[4]Present address: Institute of Specific Prophylaxis and Tropical Medicine, Center for Pathophysiology, Infectiology and Immunology, Medical University of Vienna, Vienna, Austria.

[5]These authors contributed equally to this work

*** Correspondence:**

Wilfried Ellmeier: wilfried.ellmeier@meduniwien.ac.at

Shinya Sakaguchi: shinya.sakaguchi@meduniwien.ac.at

Supplementary Figures

Figure S1

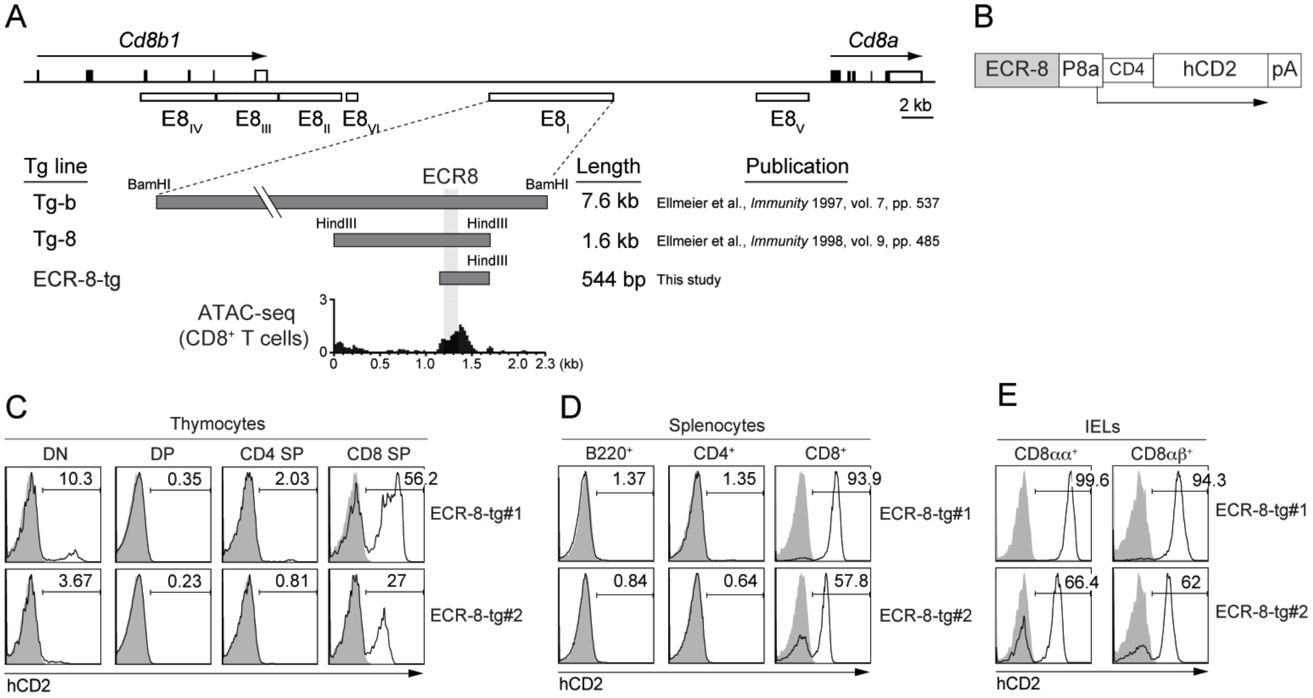

Figure S1. Generation and characterization of $E8_I$-core-transgenic (Tg) mice. **(A)** Schematic map of the *Cd8a* and *Cd8b1* gene loci (after Gorman et al. (1)). Horizontal arrows indicate the transcriptional orientation of the *Cd8a* and *Cd8b1* genes. The open bars below the *Cd8ab* gene complex indicate the location of *Cd8* enhancers $E8_I$, to $E8_V$. The lower panel indicates the transgenic constructs (including their size in kb) used to narrow down the core region of $E8_I$. The location of ECR-8 is indicated as a gray bar, and ATAC-seq peaks (in naïve $CD8^+$ T cells) surrounding ECR-8 are shown at the bottom. **(B)** Map of the transgenic constructs used to test the enhancer activity of $E8_I$-core region (2). P8a: *Cd8a* promoter, CD4: murine *Cd4* splicing module (consist of the untranslated exon I, part of intron I, and part of exon II), hCD2: human CD2 reporter gene cDNA, pA: SV40 polyadenylation sequence. **(C)** Histograms showing human CD2 (hCD2) expression on double-negative (DN), double-positive (DP), CD4 single-positive (SP) and CD8SP thymocyte subsets isolated from $E8_I$-core transgenic lines tg#1 and tg#2. **(D)** Histograms showing hCD2 expression on $B220^+$ B cells, and on $CD4^+$ and $CD8^+$ T cells ($TCR\beta^+$) isolated from the spleen of $E8_I$-core transgenic lines tg#1 and tg#2. **(E)** Histograms showing hCD2 expression in $CD8\alpha\alpha^+$ (left panels) and $CD8\alpha\beta^+$ (right panels) intraepithelial lymphocytes (IELs) isolated from the small intestine of $E8_I$-core transgenic lines tg#1 and tg#2. **(C,D,E)** Numbers in the histograms indicate the percentage of $hCD2^+$ cells. Filled histograms show a non-transgenic control. Data are representative of 6 (tg#1) and 4 (tg#2) **(C,D)** and 4 **(E)** independent experiments.

Figure S2

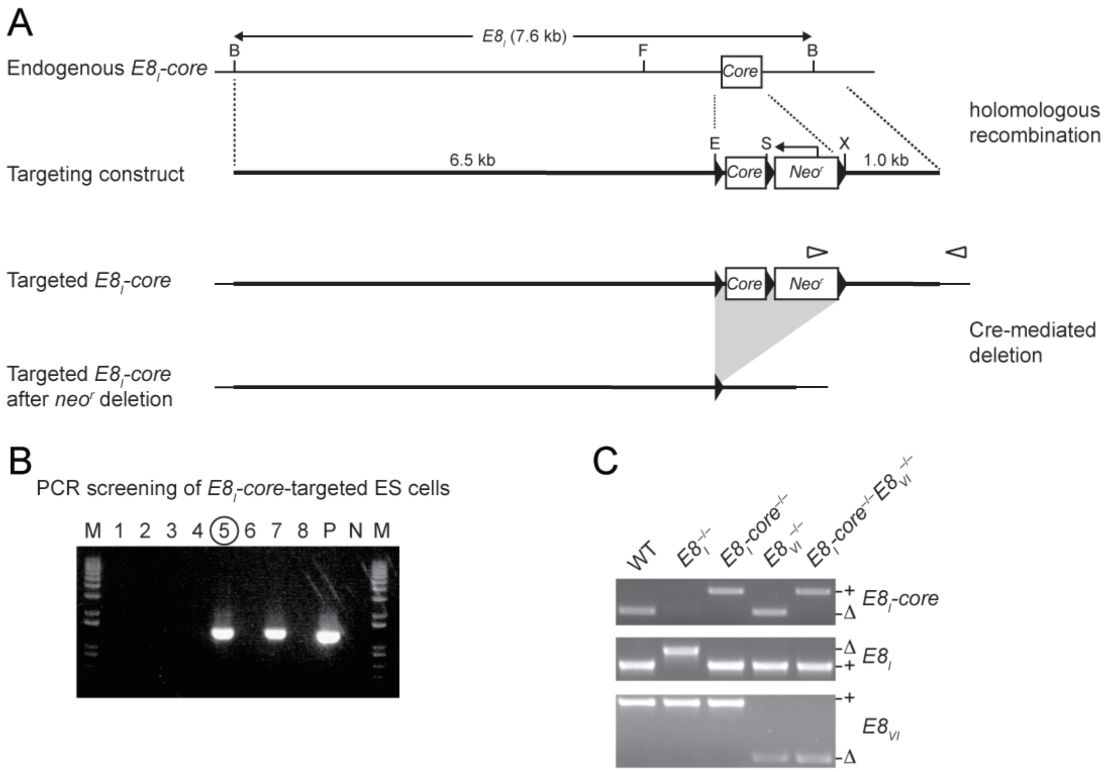

Figure S2. Generation of $E8_I$-*core*$^{-/-}$ and $E8_I$-*core*$^{-/-}E8_{VI}^{-/-}$ mice. **(A)** Targeting strategy for the generation of $E8_I$-*core*$^{-/-}$ mice. Schematic map shows the endogenous $E8_I$-*core* region (top), the targeting construct (upper middle), the targeted $E8_I$-*core* after homologous recombination (lower middle), and the targeted $E8_I$-*core* after Cre recombinase-mediated deletion of the neomycin cassette (bottom). All restriction sites of BamHI (B) and FspI (F), and EcoRI (E), SalI (S) and XhoI (X) sites important for the construction of the targeting vector are shown. Open arrowheads indicate the location of the PCR primers used for the screening of homologous recombination. The horizontal thick black lines and filled triangles indicate the arms of homology and loxP sites, respectively. *Core*, $E8_I$-*core* region; *Neo*^{r}, the neomycin cassette. **(B)** Gel image showing the PCR screening of ES clones that underwent homologous recombination. After PCR prescreening of pooled DNA samples from 8 ES clones, 8 individual clones from the positive group (indicated as 1 to 8) were further screened. No. 5 ES clone (indicated by a circle) was used for the generation of $E8_I$-*core*$^{-/-}$ mice. P, positive control for PCR; N, no template control; M, size markers. **(C)** Gel image showing genotyping PCRs to detect the $E8_I$-*core*-null (upper panel), $E8_I$-null (middle panel) and $E8_{VI}$-null (lower panel) alleles with wild-type (WT), $E8_I^{-/-}$, $E8_I$-*core*$^{-/-}$, $E8_{VI}^{-/-}$ and $E8_I$-*core*$^{-/-}E8_{VI}^{-/-}$ mouse tail samples. The sizes of PCR products corresponding to the wild-type and mutant alleles in each PCR are indicated as + and Δ, respectively. Note that there is no PCR amplification in $E8_I^{-/-}$ mouse sample, due to the deletion of the primer-binding regions. Data are representative of three independent experiments.

Figure S3

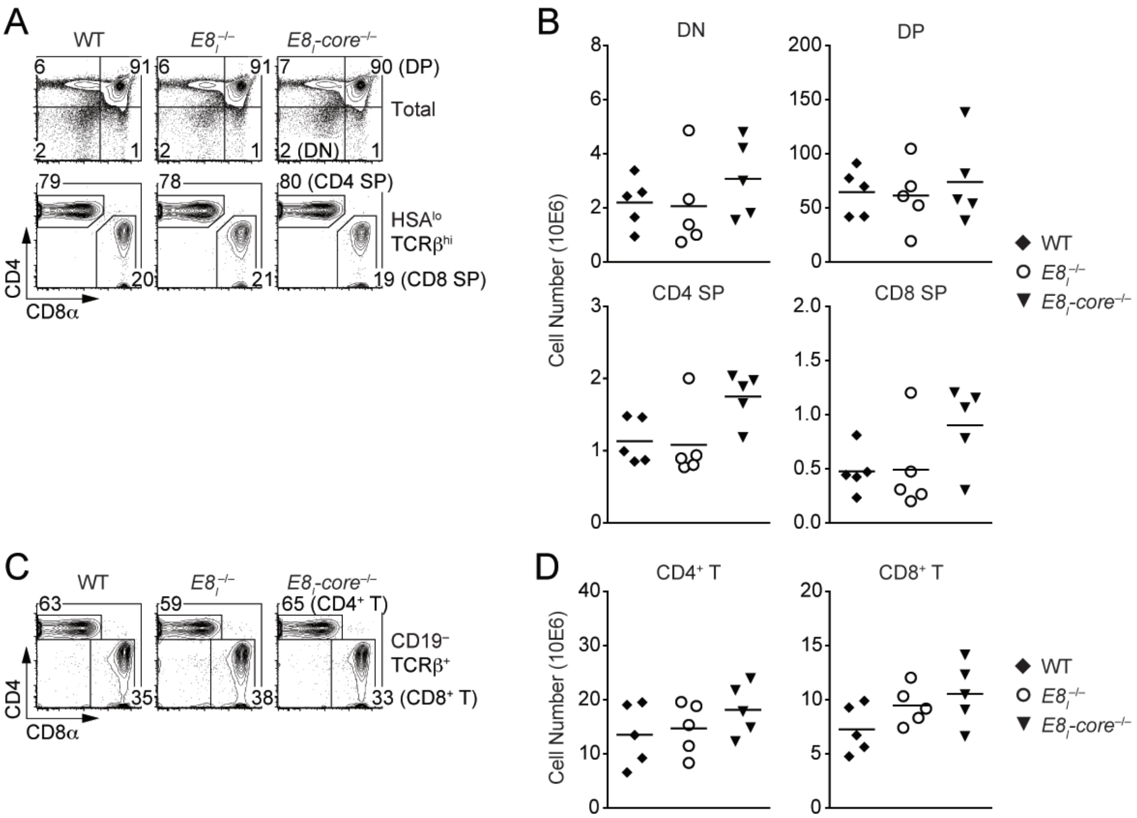

Figure S3. Characterization of $E8_{VI}^{-/-}$ mice. **(A)** Flow cytometry analysis showing CD8α and CD4 expression on total (upper panel) and HSA^{lo}TCRβ^{hi} (lower panel) thymocytes isolated from wild-type (WT), $E8_I^{-/-}$ and $E8_I$-$core^{-/-}$ mice. CD8α expression on CD8 SP thymocytes (CD8 SP) is displayed in Figure 2A. **(B)** Diagrams showing the numbers of DN (upper left), DP (upper right), CD4 SP (lower left) CD8 SP (lower right) thymocytes isolated from wild-type (WT), $E8_I^{-/-}$ and $E8_I$-$core^{-/-}$ mice. **(C)** Flow cytometry analysis showing CD8α and CD4 expression on CD19^{-}TCRβ^{+} splenocytes isolated from wild-type (WT), $E8_I^{-/-}$ and $E8_I$-$core^{-/-}$ mice. CD8α expression on CD8^{+} T cells (CD8^{+} T) is displayed in Figure 2A. **(D)** Diagrams showing the numbers of CD4^{+} (left), CD8^{+} (right) T cells isolated from wild-type (WT), $E8_I^{-/-}$ and $E8_I$-$core^{-/-}$ mice. **(A,C)** Numbers indicate the percentages within the respective regions. Data are representative of 5 mice analyzed in 5 independent experiments. **(B,D)** Each dot represents one mouse. Horizontal bars indicate mean values. A one-way ANOVA analysis followed by Tukey's multiple-comparison test was performed for statistical analysis. No comparison between two groups reached a statistically significant level (i.e. $p < 0.05$).

Figure S4

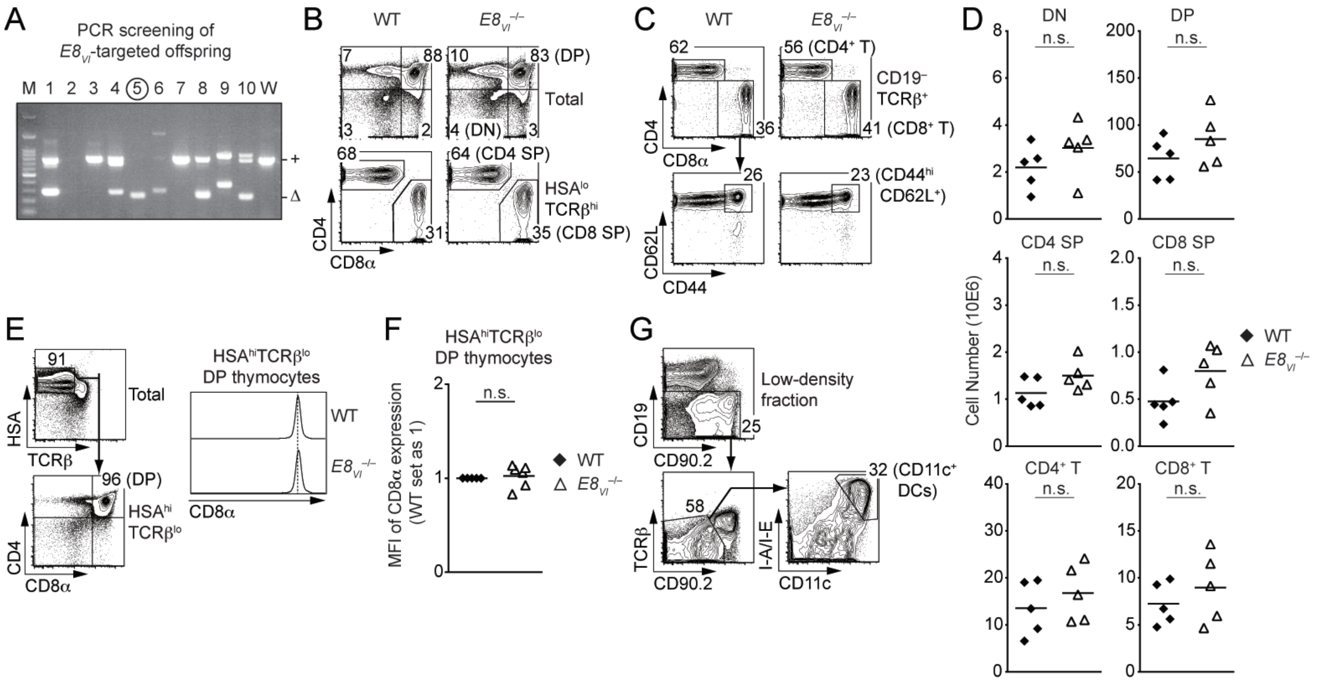

Figure S4. Generation and characterization of $E8_{VI}^{-/-}$ mice. **(A)** Gel image showing the PCR screening of $E8_{VI}$-targeted offspring obtained form the injection of guide RNAs and *Cas9* mRNA into fertilized eggs. Ten offspring (No. 1 - 10) were screened, and No. 5 offspring (indicated by a circle) was used for the establishment of the mutant strain. The sizes of PCR products derived from wild-type and $E8_{VI}$-*core*-null alleles were indicated as + and Δ, respectively. W, control DNA isolated from wild-type mouse; M, size markers. **(B)** Flow cytometry analysis showing CD8α and CD4 expression on total (upper panel) and $HSA^{lo}TCR\beta^{hi}$ (lower panel) thymocytes isolated from wild-type (WT) and $E8_{VI}^{-/-}$ mice. CD8α expression on CD8 SP thymocytes (CD8 SP) is displayed in Figure 3A. **(C)** Flow cytometry analysis showing CD8α and CD4 expression on $CD19^{-}TCR\beta^{+}$ splenocytes (upper panel) and CD62L and CD44 expression on $CD19^{-}TCR\beta^{+}CD4^{-}CD8\alpha^{+}$ splenocytes (lower panel) isolated from wild-type (WT) and $E8_{VI}^{-/-}$ mice. CD8α expression on $CD8^{+}$ T cells ($CD8^{+}$ T) and $CD8^{+}CD44^{hi}CD62L^{+}$ T cells ($CD44^{hi}CD62L^{+}$) is displayed in Figure 3A. **(D)** Diagrams showing the numbers of DN (upper left), DP (upper right), CD4 SP (middle left) and CD8 SP (middle right) thymocytes as well as $CD4^{+}$ (lower left) and $CD8^{+}$ (lower right) T cells isolated from wild-type (WT) and $E8_{VI}^{-/-}$ mice. **(E)** Representative gating strategy for the analysis of CD8α expression on $HSA^{hi}TCR\beta^{lo}$ DP thymocytes (left panel) and histograms showing CD8α expression on $HSA^{hi}TCR\beta^{lo}$ DP thymocytes isolated from wild-type (WT) and $E8_{VI}^{-/-}$ mice (right panel). **(F)** Diagrams showing the relative mean fluorescence intensity (MFI) of CD8α expression on $HSA^{hi}TCR\beta^{lo}$DP thymocytes isolated from wild-type (WT) and $E8_{VI}^{-/-}$ mice. **(G)** Representative gating strategy for the analysis of CD8α expression on DCs. CD4 and CD8α expression on $CD11c^{+}$ dendritic cells (DCs) is displayed in Figure 3C. **(B,C,E,G)** Numbers indicate the percentages within the respective regions. Data are representative of 5 mice analyzed in 5 independent experiments. **(D,F)** Each dot represents one mouse. Horizontal bars indicate mean values. An unpaired Student's *t*-test **(D)** and a one-sample *t*-test **(F)** were performed for statistical analysis. "n.s.", not significant.

Figure S5

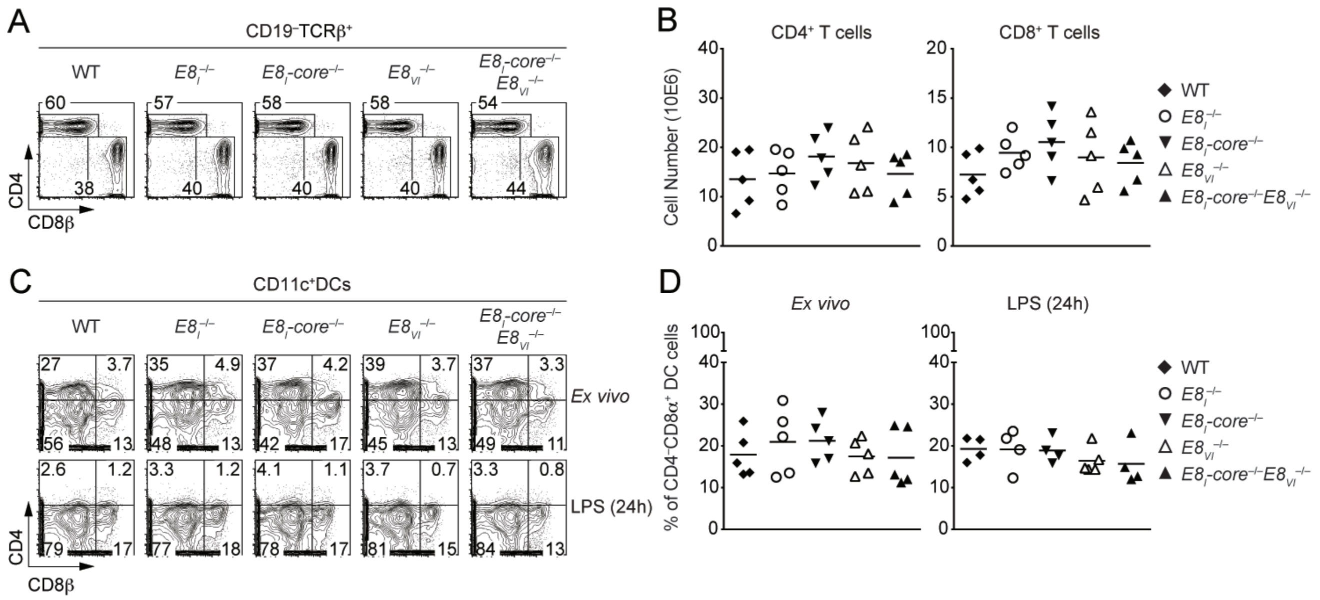

Figure S5. Characterization of $E8_I\text{-}core^{-/-}$ and $E8_I\text{-}core^{-/-}E8_{VI}^{-/-}$ mice. **(A)** Flow cytometry analysis showing CD8α and CD4 expression on CD19⁻TCRβ⁺ splenocytes isolated from from wild-type (WT), $E8_I^{-/-}$, $E8_I\text{-}core^{-/-}$, $E8_{VI}^{-/-}$ and $E8_I\text{-}core^{-/-}E8_{VI}^{-/-}$ mice. **(B)** Diagrams showing the numbers of splenic $CD4^+$ and $CD8^+$ T cells isolated from wild-type (WT), $E8_I^{-/-}$, $E8_I\text{-}core^{-/-}$, $E8_{VI}^{-/-}$ and $E8_I\text{-}core^{-/-}E8_{VI}^{-/-}$ mice. **(C)** Flow cytometry analysis showing CD8α and CD4 expression on wild-type (WT), $E8_I^{-/-}$, $E8_I\text{-}core^{-/-}$, $E8_{VI}^{-/-}$ and $E8_I\text{-}core^{-/-}E8_{VI}^{-/-}$ splenic dendritic cells (DCs), which were either freshly isolated (upper panel) or stimulated with LPS for 24 hours (lower panel). The gating strategy for DCs is shown in Figure S4G. **(D)** Diagrams showing the percentage of the $CD4^-CD8\alpha^+$ population within wild-type (WT), $E8_I^{-/-}$, $E8_I\text{-}core^{-/-}$, $E8_{VI}^{-/-}$ and $E8_I\text{-}core^{-/-}E8_{VI}^{-/-}$ splenic dendritic cells (DCs), which were either freshly isolated (left) or stimulated with LPS for 24 hours (right). **(A,C)** Numbers indicate the percentages within the respective regions. Data are representative of 5 mice analyzed in 5 independent experiments. **(B,D)** Each dot represents one mouse. Horizontal bars indicate mean values. A one-way ANOVA analysis followed by Tukey's multiple-comparison test was performed for statistical analysis. No comparison between two groups reached a statistically significant level (i.e. $p < 0.05$)

Figure S6

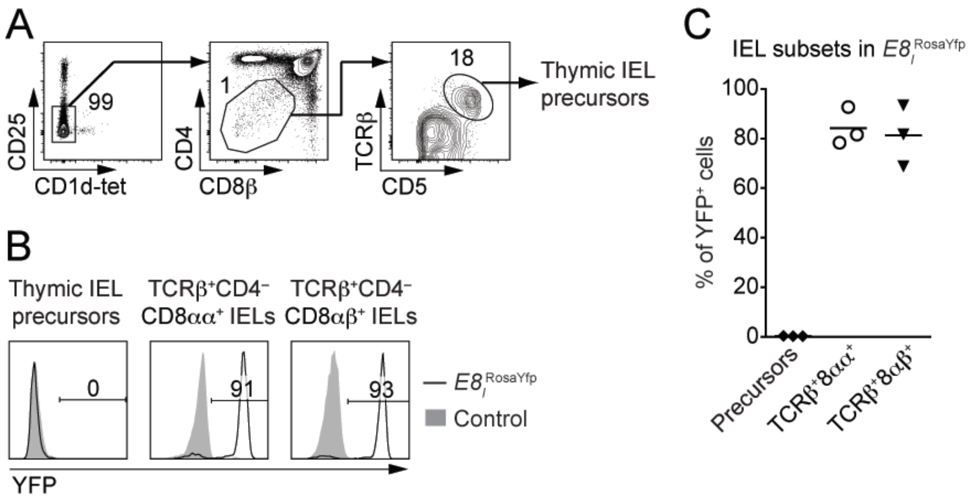

Figure S6. E8$_I$ is not active in thymic IEL precursors. **(A)** Representative gating strategy for the analysis of E8$_I$ activity in thymic IEL precursors (defined as CD1d-tetramer (PBS57-loaded)$^-$CD25$^-$ CD4$^-$CD8$^-$TCRβ$^+$CD5$^+$). **(B)** Histograms showing YFP expression on thymic IEL precursors, TCRβ$^+$CD4$^-$CD8αα$^+$ and TCRβ$^+$CD4$^-$CD8αβ$^+$ IELs isolated from *E8$_I$*-Cre,*Rosa26*-stop-YFP (*E8$_I$*^{RosaYfp}) and *E8$_I$*-Cre control (control) mice. Numbers indicate the percentages of YFP$^+$ cells in individual *E8$_I$*^{RosaYfp} IEL subsets. **(C)** Diagram showing the percentage of the YFP$^+$ population within thymic IEL precursors (precursors), TCRβ$^+$CD4$^-$CD8αα$^+$ and TCRβ$^+$CD4$^-$CD8αβ$^+$ IELs isolated from *E8$_I$*^{RosaYfp} mice. Each dot represents one mouse. Horizontal bars indicate mean values. Data are representative of 3 mice analyzed in 3 independent experiments.

Figure S7

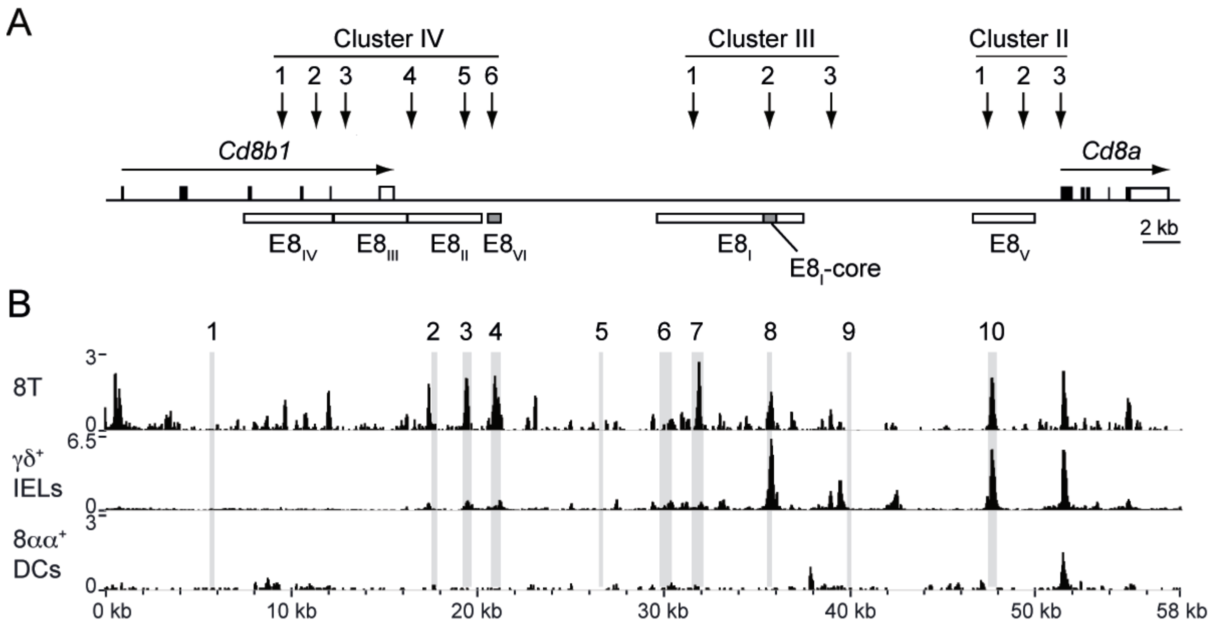

Figure S7. Chromatin accessibility at the *Cd8ab1* gene complex in TCRγδ$^+$ IELs and CD8αα$^+$ DCs. **(A)** Schematic map of the *Cd8a* and *Cd8b1* gene loci. See the figure legend of Figure 1 for further explanation. **(B)** UCSC genome browser snapshots showing the ATAC-seq signals at the *Cd8ab1* gene complex (GRCm38/mm10, chr6: 71322001-71380000) in CD8$^+$ T cells (8T), TCRγδ$^+$ IELs (γδ$^+$ IELs) and CD8αα$^+$ DCs (8αα$^+$ DCs). The ATAC-seq data of CD8$^+$ T cells and CD8αα$^+$ DCs were obtained from the Immunological Genome Project (ImmGen) (3), whereas the data of TCRγδ$^+$ IELs (isolated from conventionally-raised C57BL/6J mice) were retrieved from GSE89646 (4). Shaded bars indicate the location of ECR1 to ECR10 as previously described (5).

Supplementary Tables

Table S1. Antibodies used in this study

Antigen	Clone or Catalog number (#)	Company
CD4	RM4-5	Thermo Fisher Scientific
CD5	53-7,3	BD Biosciences
CD8α	53-6.7	Thermo Fisher Scientific
CD8β	H35-17.2	Thermo Fisher Scientific
CD11c	N418	Thermo Fisher Scientific
CD19	1D3	Biolegend
CD24	M1/69	Thermo Fisher Scientific
CD25	PC61	Biolegend
CD44	IM7	Biolegend
CD62L	MEL-14	Thermo Fisher Scientific
CD90.2	53-2.1	Biolegend
CD103	2E7	Biolegend
hCD2	LFA-2	Thermo Fisher Scientific
I-A/I-E	M5/114.15.2	Biolegend
Mouse IgG_1	RMG1-1	Biolegend
Rat IgG	#8885	Cell Signaling Technology
Runx3	R3-5G4	BD Biosciences
ThPOK	D9V5T	Cell Signaling Technology
TCRβ	H57-597	Thermo Fisher Scientific
TCRγδ	GL3	BD Biosciences

Table S2. Genomic sequence surrounding the $E8_I$-core region

Chr6: 71357410 – 71358228 (GRCm38/mm10):

TTCCCATGAGGAACAGAGCTGGAAAAGCCCATCAGAAGGGCAGCCGACAGGGAGAAGA
GACTTTTGAACAAGCCCAGCCACCCAAGGATTCACCATTCAGAGAGACCTGTCTATGTG
GGGGCTACCTCTGTCTCCCACAGCTGTGCTGGGGTCTTGTTAAAAGTGAAACTGACTTTC
AGAGGGTTCTGGGGGCGTTTGCTACAGGAAAGTCGATGTTCCACTTCAGGGTCCAACTA
GCCTCAAACAAAGCCTCTTGCTTGCTTTCAGCTTCTGGGGCCCCTTGGTTGCCTCTGCTA
CTTCTAGATGTTTCTGAGGTTACAGCAGGCCACAGCATGTTCCAGTCTAGGCAGAGGAA
ATATAAAAACCAATGCGAATGTGACTCAAGACCCCCTCGTGCTTCTACAGCACCACACA
GCTGGAAATGGCAAAGTCTGCCGGCTTCCGGGTTCCACCATTGCCGGGAAGCCACTTCC
TTTTCTCCCAACCAAATGAGATCGGCATCTGAGATCCATTCAGTAGATGAGGAATAAGA
TCAGAAAGGCTCAGGTCATACTGATCACACCGCATTAATAAAGTCCTGAGCTAGGACTC
AGCCCCAGCGGCCTGACCTGACTTAACCTATGAGTGGGATGTGACAGAGCTCAGTGGTC
CATTCACAAGCTTCATGGACGTAGAAAAGGAACCTGGGAGCAGGCCAGTGTAGCCTTAT
ACAAACCTGGGGTTATTGGATTTGAGGGGGATAAGACAACAATATAGTTCATTATAGTT
GAGTCTGTAGTTCAAAGCAGATAAAGCCAGAGAGTCAGAAGCTGAGTATGG

Underline: $E8_I$-core region

Blue: Genotyping primer-binding sequences

Table S3. Sequence of the E8$_{I}$-core-null allele in *E8$_{I}$-core$^{-/-}$* and *E8$_{I}$-core$^{-/-}$E8$_{VI}$$^{-/-}$* mice

TTCCCATGAGGAACAGAGCTGGAAAAGCCCATCAGAAGGGCAGCCGACAGGGAGAAGA
GACTTTTGAACAAGCCCAGCCACCCAAGGATTCAGGGCGAATTCATAACTTCGTATAGC
ATACATTATACGAAGTTATCTCGAGCTTCATGGACGTAGAAAAGGAACCTGGGAGCAGG
CCAGTGTAGCCTTATACAAACCTGGGGTTATTGGATTTGAGGGGGATAAGACAACAATA
TAGTTCATTATAGTTGAGTCTGTAGTTCAAAGCAGATAAAGCCAGAGAGTCAGAAGCTG
AGTATGG

Blue: Genotyping primer-binding sequences

Gray background: Insertion (i.e. a loxP site and its surrounding sequence derived from construction)

Table S4. Genomic sequence surrounding the $E8_{VI}$ region

Chr6: 71342503-71343251 (GRCm38/mm10):

CCATCAGGTACTTGGGAATGCTCAGTCTACCCAGAATAGGTGACACAAGCTTAGCGGTC
ACAGAAAGAACACACGCCCAACCATGTAGCTGTCAGTGAGCACGTGTATCCCAGCCCTG
AGCTGACATTCATGGACAACTCAAGGCCAGCACTTGCATGACTCTGAGAACAACCTGCC
CCAATGGCCCACTGGCTACTCCTTAGTCTCAGCCCTGGACAAATGGACAGAAAAATTCT
GGGATGGTATGACCTGAGGCAGAGGCAAAAATTGGCTTTGAGAAGTCTTAACAGTCTCT
CACAGGAGATTTCATGAAGGTCTCTGGGGTTGGGTGGGGATAAAGATAAGAGGGGAAG
AATTGAGCTGGTGTGGGTTAGGGGTGGAGGCCCACCTTCCCCAACACTCCCCTCCCCAG
CACCTCCAGCATCCTGTAAGCCACAAGCTACGGGCTACACAAGGCCAGGCTCAAGGCAG
GCCATGACCCCTGTGCTCCACCCTCTCTTACCACAGGCTTCTGCCACCCTCAGGGTACAC
AATGGGAAACTCGACCATCACAAAGCTCTAACTGTCTTCAAGCTTGAGCAAGAGAAGGG
GAGTCCAAGCTGACCCCTTCTTCATCTAGCACCCACCCCGAAGCCACACTGCTGCTTAAA
CTCAGACGTGCGGAAGTAGAATGGTTTCCGTAGGCAAGGAGGACTGACTAGAAGACCTT
GTATGGGTTTTCCATCCCATATCCTGTGATCTACTTTGTG

Underline: $E8_{VI}$ region

Red: Guide-RNA binding sequences

Blue: Genotyping primer-binding sequences

Table S5. Sequences of the E8$_{VI}$-null allele in *E8$_{VI}$*$^{-/-}$ and *E8$_{I}$-core*$^{-/-}$*E8$_{VI}$*$^{-/-}$ mice

E8$_{VI}$ $^{-/-}$ mice:

CCATCAGGTACTTGGGAATGCTCAGTCTACCCAGAATAGGTGACACAAGCTTAGCGGTC
ACAGAAAGAACACACGCCCAACCATGTAGCTGTCAGTGAGCACGTGTATCCCAGCCCTG
ACCTTAAACTCAGACGTGCGGAAGTAGAATGGTTTCCGTAGGCAAGGAGGACTGACTAG
AAGACCTTGTATGGGTTTTCCATCCCATATCCTGTGATCTACTTTGTG

Red: Guide RNA-binding sequences

Blue: Genotyping primer-binding sequences

Gray background: Insertions

E8$_{I}$-core$^{-/-}$*E8$_{VI}$*$^{-/-}$ mice:

CCATCAGGTACTTGGGAATGCTCAGTCTACCCAGAATAGGTGACACAAGCTTAGCGGTC
ACAGAAAGAACACACGCCCAACCATGTAGCTGTCAGGAGCACGTGTATCCCAGTTATAG
CTGCTTAAACTCAGACGTGCGGAAGTAGAATGGTTTCCGTAGGCAAGGAGGACTGACTA
GAAGACCTTGTATGGGTTTTCCATCCCATATCCTGTGTCTACTTTGTG

Red: Guide RNA-binding sequences

Blue: Genotyping primer-binding sequences

Gray background: Insertions

References

1. Gorman, S. D., Y. H. Sun, R. Zamoyska, and J. R. Parnes. Molecular linkage of the Ly-3 and Ly-2 genes. Requirement of Ly-2 for Ly-3 surface expression. *J Immunol.* (1988) 140:3646-3653.

2. Sawada, S., J. D. Scarborough, N. Killeen, and D. R. Littman. A lineage-specific transcriptional silencer regulates CD4 gene expression during T lymphocyte development. *Cell.* (1994) 77:917-929.

3. Heng, T. S., and M. W. Painter. The Immunological Genome Project: networks of gene expression in immune cells. *Nat Immunol.* (2008) 9:1091-1094. doi: 10.1038/ni1008-1091

4. Semenkovich, N. P., J. D. Planer, P. P. Ahern, N. W. Griffin, C. Y. Lin, and J. I. Gordon. Impact of the gut microbiota on enhancer accessibility in gut intraepithelial lymphocytes. *Proc Natl Acad Sci U S A.* (2016) 113:14805-14810. doi: 10.1073/pnas.1617793113

5. Sakaguchi, S., M. Hombauer, H. Hassan, H. Tanaka, N. Yasmin, Y. Naoe, et al. A novel Cd8-cis-regulatory element preferentially directs expression in CD44hiCD62L+ CD8+ T cells and in CD8alphaalpha+ dendritic cells. *J Leukoc Biol.* (2015) 97:635-644. doi: 10.1189/jlb.1HI1113-597RR
